# A randomized controlled trial-based algorithm for insulin-pump therapy in hyperglycemic patients early after kidney transplantation

**DOI:** 10.1371/journal.pone.0193569

**Published:** 2018-03-08

**Authors:** Johannes M. Werzowa, Marcus D. Säemann, Alexander Mohl, Michael Bergmann, Christopher C. Kaltenecker, Wolfgang Brozek, Andreas Thomas, Michael Haidinger, Marlies Antlanger, Johannes J. Kovarik, Chantal Kopecky, Peter X. K. Song, Klemens Budde, Julio Pascual, Manfred Hecking

**Affiliations:** 1 Department of Internal Medicine III, Clinical Division of Nephrology and Dialysis, Medical University of Vienna, Vienna, Austria; 2 Ludwig Boltzmann Institute of Osteology at the Hanusch Hospital of WGKK and AUVA Trauma Centre Meidling, 1^st^ Medical Department, Hanusch Hospital, Vienna, Austria; 3 6^th^ Medical Department, Wilhelminenspital, Vienna, Austria; 4 Faculty of Medicine, Sigmund Freud University, Vienna, Austria; 5 Medtronic GmbH, Meerbusch, Germany; 6 Department of Biostatistics, School of Public Health, University of Michigan, Ann Arbor, MI, United States of America; 7 Charité Campus Mitte, Department of Internal Medicine, Division of Nephrology, Berlin, Germany; 8 Servicio de Nefrología, Hospital del Mar-IMIM, Barcelona, Spain; Weill Cornell Medical College Qatar, QATAR

## Abstract

Treating hyperglycemia in previously non-diabetic individuals with exogenous insulin immediately after kidney transplantation reduced the odds of developing Posttransplantation Diabetes Mellitus (PTDM) in our previous proof-of-concept clinical trial. We hypothesized that insulin-pump therapy with maximal insulin dosage during the afternoon would improve glycemic control compared to basal insulin and standard-of-care. In a multi-center, randomized, controlled trial testing insulin isophane for PTDM prevention, we added a third study arm applying continuous subcutaneous insulin lispro infusion (CSII) treatment. CSII was initiated in 24 patients aged 55±12 years, without diabetes history, receiving tacrolimus. The mean daily insulin lispro dose was 9.2±5.2 IU. 2.3±1.1% of the total insulin dose were administered between 00:00 and 6:00, 19.5±11.6% between 6:00 and 12:00, 62.3±15.6% between 12:00 and 18:00 and 15.9±9.1% between 18:00 and 24:00. Additional bolus injections were necessary in five patients. Mild hypoglycemia (52–60 mg/dL) occurred in two patients. During the first post-operative week glucose control in CSII patients was overall superior compared to standard-of-care as well as once-daily insulin isophane for fasting and post-supper glucose. We present an algorithm for CSII treatment in kidney transplant recipients, demonstrating similar safety and superior short-term efficacy compared to standard-of-care and once-daily insulin isophane.

## Introduction

Posttransplantation diabetes mellitus (PTDM) is a serious and common complication after solid organ transplantation affecting a substantial proportion of kidney transplant recipients during the first year post-transplantation [1]. Although awareness of this complication has increased there still is uncertainty concerning its optimal management and prevention. Almost all patients experience hyperglycemic episodes immediately after kidney transplantation [2], mainly as a consequence of high steroid and calcineurin inhibitor doses, and due to several other reasons [3]. This vulnerable early phase may play a pivotal role in the later development of PTDM [4]. Immediate treatment of hyperglycemia using basal insulin in this early post-transplant phase has been shown to prevent later development of PTDM in our previous proof-of-concept **T**reat-to-target Trial of Basal **I**nsulin in **P**osttransplant Hyperglycemia (**TIP** study) [5]. The results of the TIP study prompted a large multi-center clinical trial aimed at testing the hypothesis that early basal insulin treatment after kidney transplantation may prevent later PTDM development (ITP-NODAT trial, ClinicalTrials.gov Identifier NCT01683331). This trial is currently enrolling patients with an intended sample-size of 300 patients in the US and Europe (NPH insulin versus conventional therapy). Exclusively at the study site Medical University of Vienna, where recruitment for the ITP-NODAT study is now completed, 28 additional patients were randomized into a third treatment group receiving continuous subcutaneous insulin infusion (CSII) with the aim to determine whether CSII is superior to basal insulin therapy for the prevention of PTDM (SAPT-NODAT study, ClinicalTrials.gov Identifier NCT01680185). Results on the primary endpoint of both trials (ITP-NODAT and SAPT-NODAT) will be published upon completion of the ITP-NODAT study.

CSII using insulin pumps has brought improvements in glycemic control and quality of life in selected patient groups suffering from T1DM and T2DM [6]. Among the advantages of CSII compared to multiple daily insulin injections (MDI) are better achievement of glycemic targets and a lower rate of severe hypoglycemic events especially in patients with unawareness of hypoglycemia [7].

CSII dosing schemes employed for patients with T1DM or advanced T2DM such as the one proposed by Renner et al. [8, 9] may not be suitable for patients with PTDM due to the different pathophysiological backgrounds of these distinct metabolic disturbances. Specifically, CSII basal rates in T1DM and T2DM are usually designed to cover about 40 to 60% of daily insulin demand with peak rates between 5:00 and 8:00. A second (smaller) peak is usually administered between 17:00 and 20:00. The remaining insulin demand is administered as prandial bolus infusions. In patients with PTDM, however, glucose levels are lowest between 2:00 and 8:00 and highest between 14:00 and 20:00 [5, 10]. Blood glucose (BG) values in the early post-operative phase are usually in the near-normal range during the second half of the night until midday reaching a maximum between 14:00 and 20:00, mainly as a consequence of the high steroid doses administered in the morning [11].

Designing a CSII algorithm based on the glucose data from our previous TIP-study, we hypothesized that administration of the maximal insulin dose during the afternoon would improve daily glucose profiles compared to basal insulin isophane and standard-of-care in kidney transplant recipients without a previous history of DM.

## Methods/Design

This study was approved by the ethics committee of the Medical University of Vienna (EK#10/2012). The clinical and research activities being reported are consistent with the Principles of the Declaration of Istanbul as outlined in the 'Declaration of Istanbul on Organ Trafficking and Transplant Tourism' and with the Declaration of Helsinki.

### Subjects

Between April 2013 and December 2015, a total of 361 patients received a kidney transplant from a deceased donor at the Medical University of Vienna. The intended (and actual) inclusion period was between the first quarter of the year 2013 and the end of the year 2015.

Non-diabetic patients who met the inclusion criteria were informed and invited to participate in either ITP-NODAT or SAPT-NODAT. Patients comfortable with both studies and willing to give informed consent for both studies were randomized 1:1:1 (basal insulin, standard-of-care control, CSII). Written informed consent was obtained before patients were transferred to the operating room. Minors or individuals unable to give informed consent were not included in the study. Trial protocols for both studies can be found online as Supporting Material (S1 Protocol and S2 Protocol).

Eighty-five patients were included in both studies (ITP-NODAT and SAPT-NODAT) at our center between April 2013 and December 2015. Twenty-eight patients were randomized into the CSII group, 26 patients into the basal insulin group and 31 patients into the control group (standard-of-care). Patients were enrolled by physicians of the study team at the acute dialysis ward before transplantation. In all the other participating centers, patients were randomized 1:1 to receive basal insulin isophane or standard of care as foreseen in the ITP-NODAT study. A unique randomization number was generated by a randomization software and assigned to each patient (patient randomization number). Sealed envelopes containing the randomization group were used for each participant/randomization number. The envelopes were provided by a person not involved in the study (clinical study support of the Medical University of Vienna).

Inclusion criteria were age >18 years, no history of diabetes mellitus before transplantation, and treatment with a standard immunosuppressive regimen consisting of once-daily tacrolimus, mycophenolate, and steroids. Exclusion criteria consisted of diagnosis of diabetes mellitus prior to kidney transplantation, receiving anti-diabetic medications, having a pre-transplant fasting glucose level ≥126 mg/dL on two occasions at least three days apart, organ transplant other than kidney, history of more than one organ transplantation in the past, therapy with an unlicensed drug or therapy within one month prior to study entry, history of hypersensitivity to injectable insulin, and documented HIV infection.

### Equipment and therapies

CSII treatment was initiated on postoperative day 1 or 2 using the MiniMed Paradigm Veo^®^ Insulin Pump with the Sure-T^®^ infusion set (Medtronic, Inc, USA). Glucose self-measurements were performed at least four times per day (before breakfast, before lunch, before supper and at bedtime) by the patients with a Contour Link Glucometer (Bayer Austria GmbH) and insulin therapy was initiated when pre-supper BG reached 140 mg/dL or above. Insulin doses were slowly up-titrated starting with an initial daily insulin dose of not more than 0.1 IU/kg body weight (4–6 IU insulin lispro in most patients). The glucose measurements served to adopt the basal rates in the insulin pumps over the treatment period aiming at a pre-supper BG of 110 mg/dL. The rapid-acting insulin formulation for CSII therapy was insulin lispro (Humalog, Eli Lilly, Inc, Netherlands). The intended duration of CSII was at least fourteen days.

For CGM the iPro-2 device (Medtronic, Inc, USA) was used. CGM, however, was stopped after the inclusion of four patients due to the unavailability of study-team members during the night-time. It was therefore decided *ad hoc* to discontinue the application of the glucose sensor within the study setting.

Patients in the basal insulin group were asked to perform glucose self-measurements 3–4 times per day and once-daily basal insulin therapy was instituted in the morning (insulin isophane, Humulin^®^, Eli Lilly, Inc, Netherlands) in patients with pre-supper BG exceeding 139 mg/dL with a pre-supper BG target of 110 mg/dL.

Patients in the control group received no routine glycemia monitoring besides once daily fasting BG measurements in the morning. During the first post-operative week 4 times daily capillary BG measurements were performed in most patients following the standard of the ward. Patients whose glucose values exceeded 200 mg/dL were subsequently monitored and, if sustained hyperglycemia was confirmed, covered by short-acting insulin lispro (Humalog^®^, Eli Lilly, Inc, Netherlands) aiming at pre-lunch and pre-supper BG values below 200 mg/dL. In case a permanent antidiabetic medication became necessary, sulfonylureas were the treatment of choice (standard-of-care).

Immunosuppressive therapy was uniform in all patients and consisted of an IL2-receptor antibody (basiliximab 20 mg) prior to transplantation and on day 4 post transplantation, steroids (at transplantation: methylprednisolone 500 mg iv; day 1: prednisone 200 mg iv, day 2: 80 mg iv, day 3: 60 mg po, day 4: 40 mg po, day 5–14: 20 mg po, day 15–28: 15 mg po), mycophenolate-mofetil and once-daily tacrolimus to maintain a trough level of 8–12 ng/mL.

### Outcomes

The primary outcome of the SAPT-NODAT study is the level of HbA1c three months after transplantation and will be published after completion of both studies (SAPT-NODAT and ITP-NODAT). This manuscript describes the glycemic control and safety regarding hypoglycemic events in all three groups during the early post-operative period. BG measurements for all three groups are available for the first post-operative week. Follow-up including secondary endpoints will be completed 24 months after transplantation.

### Sample size calculation

Based on a two-sided testing and an expected change in HbA1c of 10% with a standard deviation of 17%, an α = 0.05 and a ß = 0.2, a minimum sample size of 25 patients per group was determined (primary outcome, not presented in this manuscript). For details see Supporting Files S1 Protocol, S2 Protocol and S1 Checklist (accessible online).

### Statistical analysis

Data were displayed descriptively as proportions for categorical variables or means ± standard deviations for numerical variables. For differences in baseline characteristics between the groups 2-way analysis of variance (ANOVA) was used for numerical parameters nominal parameters and Pearson’s Chi-squared tests for categorical parameters. 2-way analysis of variance (ANOVA) served to evaluate group differences in BG levels along the time axis, with Bonferroni post-hoc correction applied to group comparisons. Applying a confidence level of 95% throughout, differences were considered statistically significant at *p*<0.05. All statistical analyses were conducted in SPSS, version 19 (SPSS Inc., Chicago, Illinois).

## Results and discussion

**Fig 1:**
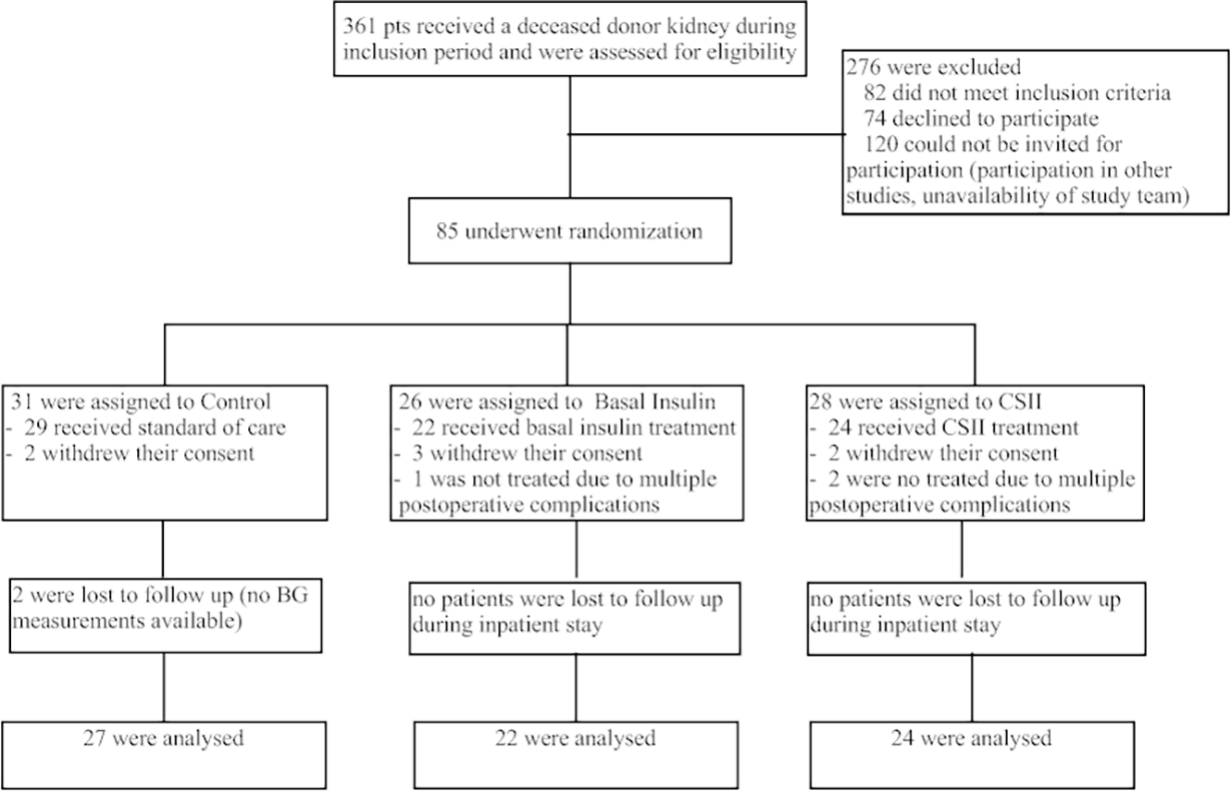
Enrollment and randomization of the SAPT-NODAT and ITP-NODAT trial participants at the Medical University of Vienna.

Twenty-eight fully consented individuals were randomized into the CSII group immediately before kidney transplantation (study flow chart: Fig 1). Table 1 depicts the baseline characteristics of all three groups. There were no significant differences between the groups regarding age, sex, BMI, number of transplant, HbA1c, native kidney disease, hepatitis C and CMV status, immunosuppression, lipid levels, comorbidities and blood pressure. All participants developed pre-supper BG levels above 140 mg/dL during the first 3 days after transplantation. Two patients in the CSII group withdrew their consent after transplantation prior to the initiation of CSII therapy and two patients experienced multiple surgical complications immediately after transplantation requiring further surgical interventions with prolonged intensive care stay. Thus, these two patients did not receive CSII therapy. In the remaining 24 participants CSII therapy was established no later than by postoperative day 3 for a mean duration of 19.5 days (range 2–81 days). Five patients had CSII therapy for less than 14 days. Reasons for premature removal (before day 14) of the CSII device were: dismissal from the ward owing to excellent graft function in two patients; and refusal to continue CSII therapy in three patients.

**Table 1:** Baseline patient characteristics.

	Control	Basal insulin	Insulin pump	p
	(n = 27)	(n = 22)	(n = 24)	
Men	15 (56%)	14 (63%)	17 (71%)	n.s.
Mean age (yr)	52.9±13.4	53.9±12.7	52.9±11.8	n.s.
1st KTX	23 (85%)	17 (77%)	19 (79%)	n.s.
2nd KTX	4 (15%)	5 (23%)	5 (21%)	n.s.
Mean BMI (kg/m^2^)	26.4±6.3	27.1±5.4	26.1±4.9	n.s.
HbA1c (%)	5.2±0.6	5.0±0.4	5.2±0.4	n.s.
Hepatitis C	4 (15%)	0 (0%)	1 (4%)	n.s.
CMV intermediate risk	16 (59%)	12 (55%)	16 (67%)	n.s.
CMV high risk	5 (19%)	7 (32%)	6 (25%)	n.s.
PRA highest (≥10%)	2 (7%)	2 (9%)	3 (13%)	n.s.
Mean TAC trough level (day 7; ng/mL)	8.8±4.2	7.7±3.0	8.3±3.0	n.s.
Mean prednisone dose (day 7; mg)	21±5	21±4	23±7	n.s.
Mean MMF dose (day 7; mg)	1889±482	1952±590	1762±539	n.s.
Native kidney disease				
glomerular disease	12 (44%)	4 (18%)	7 (29%)	n.s.
vascular disease	6 (22%)	7 (32%)	4 (17%)	n.s.
polycystic disease	2 (7%)	5 (23%)	3 (13%)	n.s.
urologic disease	0 (0%)	0 (0%)	1 (4%)	n.s.
tubulointerstitial disease	1 (4%)	0 (0%)	1 (4%)	n.s.
unknown	6 (22%)	6 (27%)	8 (17%)	n.s.
Comorbid conditions				
cardiovascular	10 (37%)	10 (45%)	12 (50%)	n.s.
respiratory	0 (0%)	4 (18%)	4 (17%)	n.s.
urinary	1 (4%)	1 (5%)	2 (8%)	n.s.
neurologic	0 (0%)	2 (9%)	0 (0%)	n.s.
endocrinologic	3 (11%)	1 (5%)	4 (17%)	n.s.
Lipid levels (mg/dL)				
Total cholesterol	169±44	168±37	177±44	n.s.
LDL cholesterol	94±40	96±31	95±36	n.s.
HDL cholesterol	50±18	52±18	44±15	n.s.
Triglycerides	140±74	160±76	188±144	n.s.
Blood pressure (mm Hg)				
Systolic	137±23	135±28	138±17	n.s.
Diastolic	76±23	83±18	81±11	n.s.

Data are presented as means ±SD or frequencies and percentages.

CSII therapy was initiated at the earliest pre-supper BG measurement surpassing 140 mg/dL. Using the glucose self-measurements, the basal rates were adapted over the next days and a stable insulin dose was reached at 7±4 days after CSII therapy initiation in all patients. Additional bolus injections before the meals were usually avoided to keep the handling of the pumps as simple as possible. However, five patients with insufficiently controlled blood glucose were trained to administer additional insulin bolus injections before large meals aiming at postprandial values <50 mg/dL above the pre-prandial values. Mean insulin bolus dose was 3.7±2.1 IU.

**Fig 2:**
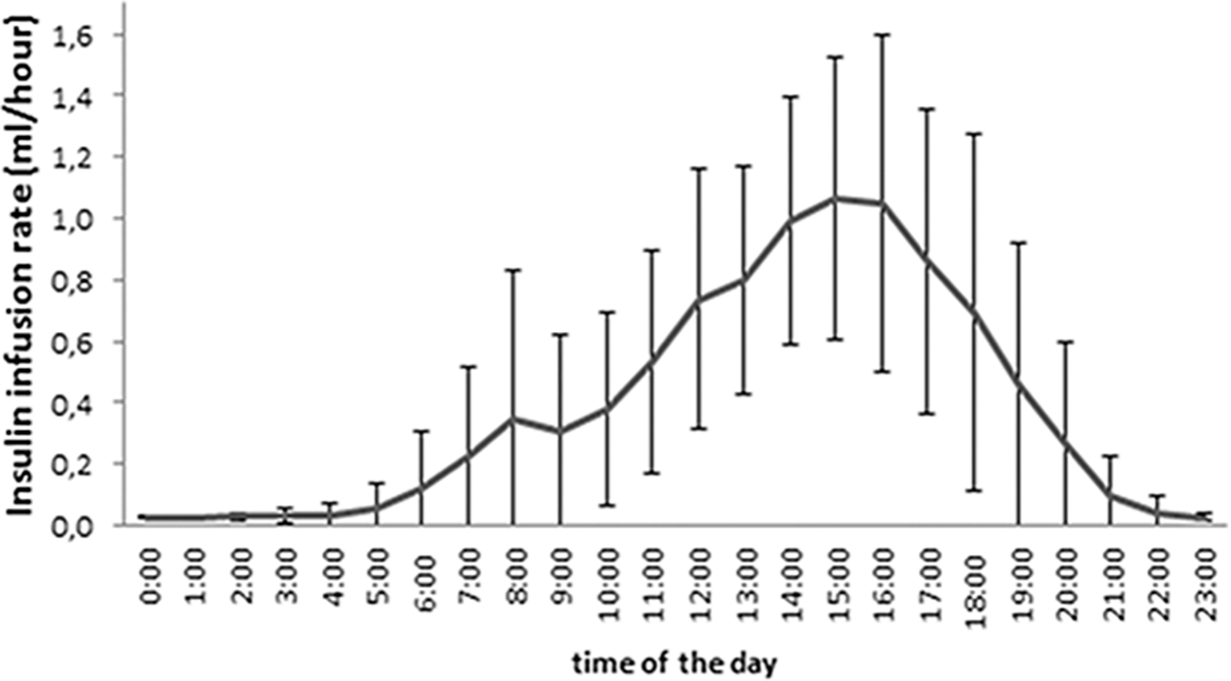
Insulin infusion rates. Mean CSII basal rate ± standard deviation after dose titration over 24 hours.

Fig 2 shows a graph of the mean stable insulin basal rates after titration by hour. The total insulin doses per hour over the entire day as well as total daily insulin doses for each individual study participant are shown in Supplemental Tables S1 Table and S2 Table. The mean total daily insulin dose in the present study amounted to 9.2±5.2 IU, which was significantly lower than the mean total insulin dose in our previous TIP study (17.0±11.4 IU per day) [5].

Of the 26 patients randomized into the basal insulin group 22 patients received basal insulin during the first post-operative week (3 patients withdrew their consent before insulin therapy was instituted and one patient did not receive basal insulin due to multiple postoperative complications). The mean insulin dose in the basal insulin group at post-operative day 7 was 11.7±6.2 IU of insulin isophane.

Thirty-one patients were assigned to the control group and BG measurements during the first post-operative week were available for 27 patients (two patients withdrew their consent and in two patients no glucometer measurements were available). During the first week antihyperglycemic therapy was instituted in 9 patients in the control group (8 patients received short acting insulin between 4 and 12 IU/day and one patient received 60 mg gliclazid per day).

**Fig 3:**
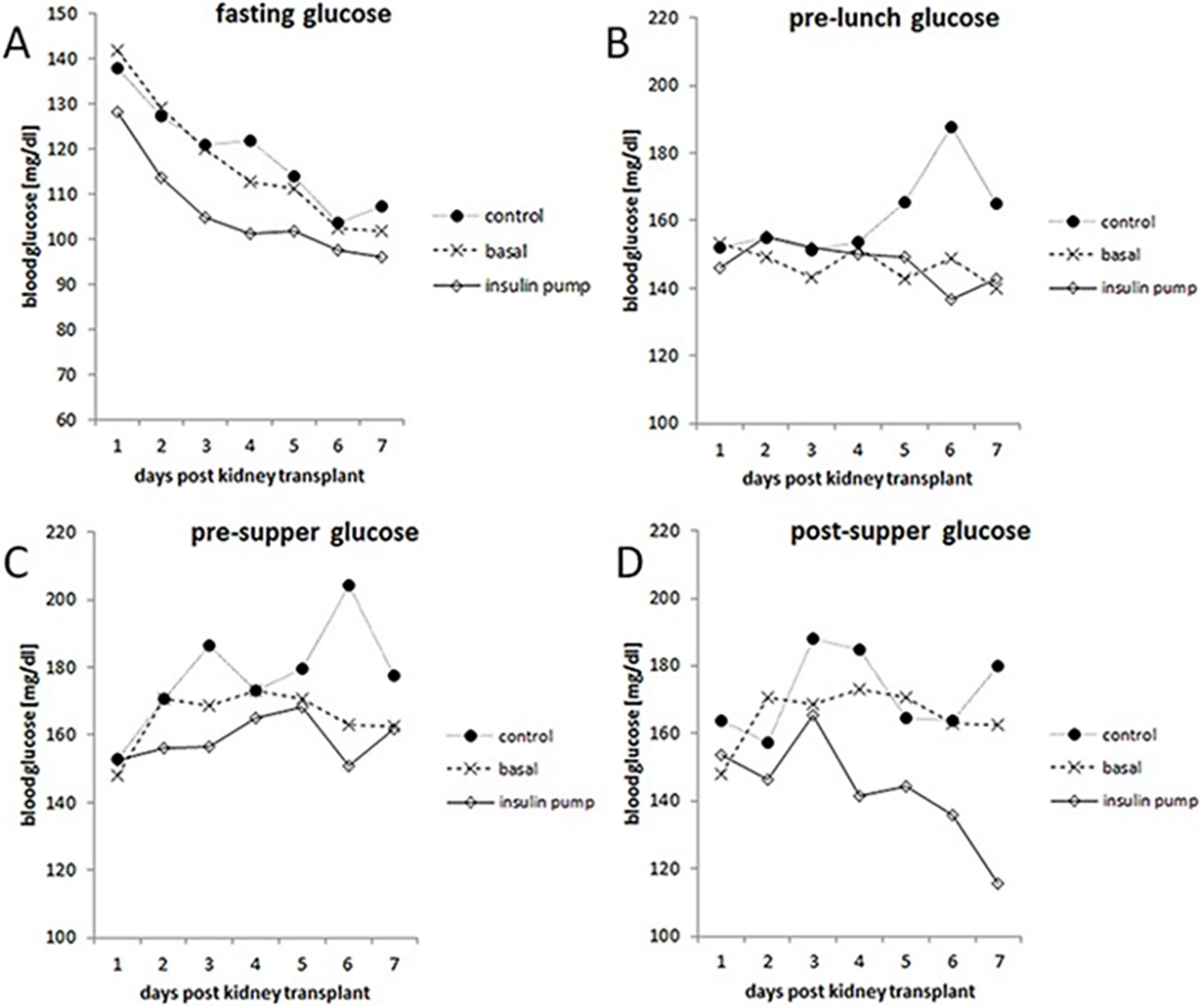
Mean BG profiles of patients under CSII treatment, basal insulin isophane, and standard-of-care over 7 days after transplantation. CSII patients had significantly lower BG compared to the control group for all times of the day except for pre-lunch and compared to the basal insulin group for fasting and post-supper glucose (2-way ANOVA, Bonferroni post-hoc test): (A) fasting, p<0.0001 vs. control and vs. basal insulin; 5% (CSII group) 8% (basal insulin group) and 34% (control group) of BG results were missing. (B) pre-lunch; 25% (CSII group) 14% (basal insulin group) and 49% (control group) of BG results were missing. (C) pre-supper, p = 0.009 vs. control; 10% (CSII group) 9% (basal insulin group) and 49% (control group) of BG results were missing. (D) post-supper, p<0.0001 vs. control and p = 0.004 vs. basal insulin; 12% (CSII group) 24% (basal insulin group) and 68% (control group) of BG results were missing.

Fig 3 shows the results of BG measurements for all three groups at the 4 available time-points (fasting, pre-lunch, pre-supper, post-supper) during the first post-operative week. BG levels in CSII patients were significantly lower compared to basal insulin and control for fasting glucose and post-supper BG and significantly lower compared to control but not to basal insulin for pre-supper BG. For legibility standard deviations were not included in the graph. Glucose measurement data including standard deviations for all three groups can be found in Supplemental S3 Table.

Table 2 shows a summary of adverse events related to antihyperglycemic therapy over the first three weeks post-transplantation for the basal insulin and the CSII group. Hypoglycemic events with a measured BG value of 52, respectively 60 mg/dL occurred in 2 patients during CSII therapy. One patient (the one with BG 60 mg/dL) noticed the event as dizziness, which disappeared after ingestion of glucose-containing food, the other patient did not notice the event. No patient experienced more than one hypoglycemic event. Six patients did not feel comfortable with the device or even experienced local pain and skin reactions that subsided after device removal. In 3 patients the device had to be removed on patients’ request. Two patients felt very comfortable with the device and continued CSII therapy after hospital discharge for a total duration of 81 and 53 days, respectively. In the basal insulin group, no hypoglycemic events were observed during the inpatient stay.

**Table 2:** Adverse events, insulin therapy-related only.

	Basal insulin	Insulin pump
	(n = 22)	(n = 24)
symptomatic hypoglycemia	0	1 (4%)
asymptomatic hypoglycemia	0	1 (4%)
local reaction/pain	0	4 (16%)
general discomfort with device	n/a	2 (8%)

Hypoglycemia was defined as BG < 60 mg/dL.

In the present study, CSII was used for the first time against hyperglycemia in patients immediately after kidney transplantation. Based on PTDM physiology, we were able to develop an hourly CSII dosing scheme which was well tolerated and resulted in rapid and adequate glycemic control postoperatively. BG levels in CSII-treated patients were, during the first week, significantly lower compared to the basal insulin isophane group and to the standard-of-care control group for most time points. The highest relative reduction was seen post-supper. This might indicate that constant delivery of short-acting insulin is more effective for the treatment of postprandial BG excursions than once-daily injection of basal insulin.

The mean daily insulin dose of about 9 IU which was administered in CSII-patients was lower than the mean daily insulin dose of about 12 IU in the basal insulin isophane group. In the previous TIP study, the basal insulin dose was even 17 IU/day. These are not unexpected findings, as insulin demand has been shown to be lower with CSII therapy, compared to conventional therapy in patients with T1 and T2DM [12, 13].

Insulin pump therapy and most recently even closed loop insulin delivery systems (artificial pancreas) receive increasing attention in the management of insulin-dependent diabetics, and these technologies hold promise as innovative therapy of T1DM [14, 15]. The insulin pump algorithm presented here was designed for previously nondiabetic patients who require treatment of posttransplant hyperglycemia, and not for patients with pre-existing T1 or T2DM.

In our study, hypoglycemic events occurred twice during the postoperative period in the CSII group, in comparison to five asymptomatic episodes of hypoglycaemia in the previous TIP study basal insulin isophane group. This rate was lower than we expected, and justifies our careful approach, retrospectively. While it would have been daring to develop the described algorithm for patients receiving steroids outside of the hospital setting, we would neither consider it impossible nor dangerous to send patients home with a functioning insulin pump, even if they have much shorter postoperative hospitalization periods (in the US, for example) than patients transplanted in most of Europe. At our center patients usually remain hospitalized for a period of no less than two weeks after kidney transplantation, whereas in US transplant centers, patients are more often dismissed after 4 days. CSII therapy would therefore have to be continued on an outpatient basis in most US centers. If CSII may not be continued outside of the hospital in patients who still need insulin, therapy can likely be converted to a basal insulin regimen. To avoid poor therapy adherence, we advise that CSII be instituted in patients who are well informed about the advantages and disadvantages of pump-therapy, and who are able to take an active decision for this form of therapy.

This study has some limitations: Not all patients adhered to the four times daily glucose self-measurements and therefore glucose data are missing. In the control group the rate of missing values is particularly high because these patients were not trained to perform measurements themselves. The present analysis of SAPT-NODAT trial data does not present results on the primary endpoint, and the statistical examinations were not pre-defined prior to study start. We therefore stress that our determinations of statistical significance (p-values) were exploratory in nature. This limitation, however, does not concern the principal message of the present study, as the algorithm for CSII therapy was derived descriptively. It is possible that we underestimate the true number of hypoglycemic events under CSII therapy, because we do not have data on glucose values during the night-time (especially between 0:00 and 6:00 AM). Clinically, no symptoms were reported that would point towards severe hypoglycemic events during night-time.

Data on the glucose effects in the three SAPT/ITP-NODAT groups were compared only for the first post-operative week to demonstrate feasibility and short-term efficacy. In addition, BG measurements for all three groups were only available during the first post-operative week.

## Conclusions

CSII therapy following the presented algorithm is easily feasible and safe in patients with hyperglycemia immediately after kidney transplantation, clearly improving glycemia over the first week of therapy even when compared to basal insulin isophane treatment. Whether CSII therapy can also help prevent the development of PTDM awaits completion of the ongoing SAPT-NODAT trial follow-up.

## Supporting information

S1 Checklist CONSORT 2010 checklist. Accessible online.
(DOC)

S1 Table Individual insulin lispro doses (IU) over the day (0:00 to 13:00) and mean doses over all patients (IU and % of total daily dose). (DOCX)

S2 Table Individual insulin lispro doses (IU) over the day (13:00 to 23:00), total doses per day and mean doses over all patients (IU and % of total daily dose); final doses after titration and duration of titration phase (days). (DOCX)

S3 Table Mean serum glucose (mg/dL) over 7 days including standard deviation (SD). (DOCX)

S1 Protocol ITP-NODAT Protocol. **Study protocol.** Accessible online.
(PDF)

S2 Protocol SAPT-NODAT Protocol. **Study protocol**. Accessible online.
(PDF)

## Abbreviations

BG: blood glucose
BMI: body mass index
CMV: cytomegalovirus
CSII: continuous subcutaneous insulin infusion
HbA1c: haemoglobin A1c
HDL: high-density lipoprotein
IU: international units
KTX: kidney transplantation
LDL: low-density lipoprotein
MDI: multiple daily insulin injections
MMF: mycophenolate mofetil
PRA: panel-reactive antibodies
PTDM: posttransplantation diabetes mellitus
TAC: tacrolimus
T1DM: type 1 Diabetes mellitus
T2DM: type 2 Diabetes mellitus
