# Supplementary material for: A randomized controlled trial-based algorithm for insulin-pump therapy in hyperglycemic patients early after kidney transplantation
Source: PLoS One. 2018 Mar 8;13(3):e0193569. doi: 10.1371/journal.pone.0193569 (PMC5843249; doi:10.1371/journal.pone.0193569)
Supplement: S1 Table — (DOCX) [file pone.0193569.s002.docx]

**S1 Table. Individual insulin lispro doses (IU) over the day (0:00 to 13:00) and mean doses in all patients (IU and % of total daily dose); final dose after titration.**

|  | **0:00** | **1:00** | **2:00** | **3:00** | **4:00** | **5:00** | **6:00** | **7:00** | **8:00** | **9:00** | **10:00** | **11:00** | **12:00** | **13:00** |
| --- | --- | --- | --- | --- | --- | --- | --- | --- | --- | --- | --- | --- | --- | --- |
| Patient 1 | 0.03 | 0.03 | 0.03 | 0.03 | 0.03 | 0.03 | 0.10 | 0.10 | 0.40 | 0.45 | 0.60 | 0.70 | 0.80 | 1.00 |
| Patient 2 | 0.03 | 0.03 | 0.03 | 0.03 | 0.03 | 0.03 | 0.03 | 0.45 | 0.75 | 0.80 | 0.85 | 1.10 | 1.10 | 1.10 |
| Patient 3 | 0.03 | 0.03 | 0.03 | 0.03 | 0.03 | 0.20 | 0.08 | 1.00 | 1.25 | 1.50 | 1.50 | 1.75 | 2.00 | 2.00 |
| Patient 4 | 0.03 | 0.03 | 0.03 | 0.03 | 0.03 | 0.03 | 0.03 | 0.05 | 0.08 | 0.10 | 0.20 | 0.30 | 0.40 | 0.60 |
| Patient 5 | 0.03 | 0.03 | 0.03 | 0.03 | 0.03 | 0.03 | 0.05 | 0.05 | 0.05 | 0.05 | 0.05 | 0.10 | 0.20 | 0.40 |
| Patient 6 | 0.03 | 0.03 | 0.03 | 0.03 | 0.03 | 0.03 | 0.05 | 0.10 | 0.13 | 0.13 | 0.20 | 0.30 | 0.40 | 0.60 |
| Patient 7 | 0.03 | 0.03 | 0.03 | 0.03 | 0.03 | 0.10 | 0.20 | 0.03 | 0.30 | 0.50 | 0.50 | 0.50 | 0.90 | 0.90 |
| Patient 8 | 0.03 | 0.03 | 0.03 | 0.03 | 0.03 | 0.03 | 0.03 | 0.10 | 0.20 | 0.20 | 0.20 | 0.30 | 0.60 | 0.60 |
| Patient 9 | 0.03 | 0.03 | 0.03 | 0.03 | 0.03 | 0.03 | 0.05 | 0.05 | 0.05 | 0.10 | 0.20 | 0.40 | 0.50 | 0.60 |
| Patient 10 | 0.03 | 0.03 | 0.03 | 0.03 | 0.03 | 0.03 | 0.03 | 1.10 | 2.20 | 0.20 | 0.20 | 0.20 | 1.70 | 0.70 |
| Patient 11 | 0.03 | 0.03 | 0.03 | 0.03 | 0.03 | 0.03 | 0.03 | 0.20 | 0.50 | 0.20 | 0.20 | 1.00 | 0.70 | 0.70 |
| Patient 12 | 0.03 | 0.03 | 0.03 | 0.03 | 0.03 | 0.03 | 0.05 | 0.08 | 0.10 | 0.20 | 0.40 | 0.60 | 0.80 | 0.80 |
| Patient 13 | 0.03 | 0.03 | 0.03 | 0.03 | 0.03 | 0.03 | 0.08 | 0.13 | 0.18 | 0.20 | 0.20 | 0.30 | 0.35 | 0.40 |
| Patient 14 | 0.03 | 0.03 | 0.03 | 0.03 | 0.03 | 0.03 | 0.08 | 0.13 | 0.18 | 0.20 | 0.20 | 0.30 | 0.50 | 0.70 |
| Patient 15 | 0.03 | 0.03 | 0.03 | 0.03 | 0.03 | 0.03 | 0.08 | 0.13 | 0.18 | 0.20 | 0.20 | 0.30 | 0.35 | 0.40 |
| Patient 16 | 0.03 | 0.03 | 0.03 | 0.03 | 0.03 | 0.03 | 0.08 | 0.08 | 0.10 | 0.30 | 0,50 | 0.70 | 0.90 | 1.10 |
| Patient 17 | 0.03 | 0.03 | 0.03 | 0.03 | 0.05 | 0.05 | 0.08 | 0.08 | 0.10 | 0.20 | 0.60 | 0.60 | 0.80 | 0.90 |
| Patient 18 | 0.03 | 0.03 | 0.03 | 0.03 | 0.03 | 0.03 | 0.05 | 0.05 | 0.10 | 0.20 | 0.30 | 0.40 | 0.50 | 0.70 |
| Patient 19 | 0.03 | 0.03 | 0.03 | 0.03 | 0.03 | 0.03 | 0.08 | 0.10 | 0.10 | 0.13 | 0.15 | 0.50 | 0.80 | 0.80 |
| Patient 20 | 0.03 | 0.03 | 0.03 | 0.03 | 0.03 | 0.03 | 0.03 | 0.05 | 0.08 | 0.10 | 0.30 | 0.40 | 0.50 | 0.50 |
| Patient 21 | 0.03 | 0.03 | 0.03 | 0.03 | 0.03 | 0.03 | 0.05 | 0.05 | 0.10 | 0.20 | 0.30 | 0.40 | 0.50 | 0.70 |
| Patient 22 | 0.03 | 0.03 | 0.03 | 0.03 | 0.03 | 0.05 | 0.08 | 0.13 | 0.15 | 0.15 | 0.30 | 0.50 | 0.70 | 0.90 |
| Patient 23 | 0.05 | 0.05 | 0.10 | 0.15 | 0.20 | 0.40 | 0.60 | 0.70 | 0.80 | 0.80 | 0.80 | 0.90 | 1.20 | 1.60 |
| Patient 24 | 0.03 | 0.03 | 0.03 | 0.03 | 0.03 | 0.08 | 0.10 | 0.13 | 0.15 | 0.15 | 0.20 | 0.25 | 0.50 | 0.50 |
| **MEAN (IU±SD)** | **0.0±0.0** | **0.0±0.0** | **0.0±0.0** | **0.0±0.0** | **0.0±0.0** | **0.1±0.1** | **0.1±0.2** | **0.2±0.3** | **0.3±0.5** | **0.3±0.3** | **0.4±0.3** | **0.5±0.4** | **0.7±0.4** | **0.8±0.4** |
| **MEAN (%±SD)** | **0.3±0.1** | **0.3±0.1** | **0.3±0.1** | **0.4±0.1** | **0.4±0.2** | **0.5±0.4** | **1.1±0.9** | **2.0±1.7** | **3.1±3.1** | **3.1±1.9** | **4.2±2.1** | **5.9±2.0** | **8.2±2.3** | **9.3±2.4** |
