# Supplementary material for: A randomized controlled trial-based algorithm for insulin-pump therapy in hyperglycemic patients early after kidney transplantation
Source: PLoS One. 2018 Mar 8;13(3):e0193569. doi: 10.1371/journal.pone.0193569 (PMC5843249; doi:10.1371/journal.pone.0193569)
Supplement: S2 Table — (DOCX) [file pone.0193569.s003.docx]

**S2 Table. Individual insulin lispro doses (IU) over the day (13:00 to 23:00), total doses per day and mean doses over all patients (IU and % of total daily dose); final doses after titration and duration of titration phase (days).**

|  | **13:00** | **14:00** | **15:00** | **16:00** | **17:00** | **18:00** | **19:00** | **20:00** | **21:00** | **22:00** | **23:00** | **total** | **titr. dur.** |
| --- | --- | --- | --- | --- | --- | --- | --- | --- | --- | --- | --- | --- | --- |
| Patient 1 | 1.00 | 1.10 | 1.00 | 0.90 | 0.70 | 0.50 | 0.25 | 0.10 | 0.03 | 0.03 | 0.03 | 8,9 | 4 |
| Patient 2 | 1.10 | 1.50 | 1.50 | 1.50 | 0.80 | 0.03 | 0.03 | 0.10 | 0.03 | 0.03 | 0.03 | 11,9 | 9 |
| Patient 3 | 2.00 | 2.00 | 2.00 | 2.00 | 2.00 | 2.00 | 1.50 | 1.00 | 0.20 | 0.03 | 0.03 | 24,9 | 7 |
| Patient 4 | 0.60 | 0.80 | 0.90 | 1.00 | 0.80 | 0.40 | 0.08 | 0.05 | 0.03 | 0.03 | 0.03 | 6,0 | 9 |
| Patient 5 | 0.40 | 0.60 | 0.80 | 0.80 | 0.80 | 0.60 | 0.40 | 0.20 | 0.05 | 0.03 | 0.03 | 5,4 | 5 |
| Patient 6 | 0.60 | 0.80 | 0.90 | 1.00 | 0.80 | 0.40 | 0.20 | 0.10 | 0.03 | 0.03 | 0.03 | 6,3 | 7 |
| Patient 7 | 0.90 | 0.70 | 0.50 | 0.30 | 0.20 | 0.10 | 0.03 | 0.03 | 0.03 | 0.03 | 0.03 | 6,3 | 12 |
| Patient 8 | 0.60 | 1.00 | 1.00 | 1.00 | 1.00 | 1.00 | 0.50 | 0.20 | 0.10 | 0.03 | 0.03 | 8,2 | 6 |
| Patient 9 | 0.60 | 0.70 | 1.00 | 1,20 | 1,20 | 1.00 | 0.50 | 0.10 | 0.03 | 0.03 | 0.03 | 7,9 | 12 |
| Patient 10 | 0.70 | 0.60 | 1.50 | 2,00 | 1.00 | 1.60 | 0.80 | 0.03 | 0.03 | 0.03 | 0.03 | 14,1 | 2 |
| Patient 11 | 0.70 | 1.50 | 2.00 | 2.00 | 1.60 | 1.60 | 1.50 | 1.00 | 0.20 | 0.03 | 0.03 | 15,1 | 9 |
| Patient 12 | 0.80 | 1.00 | 1.20 | 1.10 | 1.00 | 1.00 | 0.80 | 0.50 | 0.10 | 0.05 | 0.03 | 10,0 | n/a* |
| Patient 13 | 0.40 | 0.60 | 0.50 | 0.40 | 0.33 | 0.30 | 0.20 | 0.18 | 0.15 | 0.03 | 0.03 | 4,7 | 2 |
| Patient 14 | 0.70 | 0.90 | 1.00 | 1.10 | 1.00 | 0.75 | 0.50 | 0.20 | 0.05 | 0.03 | 0.03 | 8,0 | 3 |
| Patient 15 | 0.40 | 0.50 | 0.30 | 0.38 | 0.33 | 0.30 | 0.20 | 0.18 | 0.15 | 0.03 | 0.03 | 4,4 | 8 |
| Patient 16 | 1.10 | 1.50 | 1.25 | 1.00 | 0.70 | 0.08 | 0.08 | 0.03 | 0.03 | 0.03 | 0.03 | 8,62 | 5 |
| Patient 17 | 0.90 | 1.00 | 1.10 | 1.00 | 0.70 | 0.50 | 0.25 | 0.10 | 0.03 | 0.03 | 0.03 | 8,3 | 2 |
| Patient 18 | 0.70 | 0.90 | 0.70 | 0.50 | 0.40 | 0.25 | 0.20 | 0.10 | 0.05 | 0.03 | 0.03 | 5,6 | 2 |
| Patient 19 | 0.80 | 1.00 | 1.20 | 1.00 | 1.00 | 0.75 | 0.35 | 0.03 | 0.10 | 0.03 | 0.03 | 8,6 | 12 |
| Patient 20 | 0.50 | 0.60 | 0.70 | 0.60 | 0.40 | 0.20 | 0.08 | 0.05 | 0.03 | 0.03 | 0.03 | 4,8 | 12 |
| Patient 21 | 0.70 | 0.90 | 0.70 | 0.50 | 0.40 | 0.25 | 0.20 | 0.10 | 0.05 | 0.03 | 0.03 | 5,6 | 2 |
| Patient 22 | 0.90 | 1.20 | 1.00 | 0.80 | 0.60 | 0.50 | 0.40 | 0.30 | 0.20 | 0.10 | 0.03 | 8,2 | 9 |
| Patient 23 | 1.60 | 1.80 | 2.00 | 2.30 | 2.20 | 2.00 | 1.60 | 1.20 | 0.63 | 0.30 | 0.08 | 22,5 | 5 |
| Patient 24 | 0.50 | 0.60 | 0.80 | 0.80 | 0.70 | 0.50 | 0.35 | 0.20 | 0.05 | 0.03 | 0.03 | 6,2 | 6 |
| **MEAN (IU±SD)** | **0.8±0.4** | **1.0±0.4** | **1.1±0.5** | **1.0±0.6** | **0.9±0.5** | **0.7±0.6** | **0.5±0.5** | **0.3±0.3** | **0.1±0.1** | **0.0±0.1** | **0.0±0.0** | **9.2±5.2** | **7±4** |
| **MEAN (%±SD)** | **9.3±2.4** | **11.7±2.9** | **12.1±2.2** | **11.6±2.8** | **9.5±2.9** | **7.1±3.4** | **4.5±2.5** | **2.5±1.8** | **1.0±1.0** | **0.4±0.3** | **0.3±0.1** |  |  |

titr. dur. denotes titration duration, i.e. time to stable insulin rate in days for each patient; *not applicable, because pump was removed after two days of therapy
