# Supplementary material for: A randomized controlled trial-based algorithm for insulin-pump therapy in hyperglycemic patients early after kidney transplantation
Source: PLoS One. 2018 Mar 8;13(3):e0193569. doi: 10.1371/journal.pone.0193569 (PMC5843249; doi:10.1371/journal.pone.0193569)
Supplement: S3 Table — (DOCX) [file pone.0193569.s004.docx]

**S3 Table.** **Mean Serum Glucose (mg/dl) over 7 days** including standard deviation (SD)

| Mean | DAY | **1** | **2** | **3** | **4** | **5** | **6** | **7** |
| --- | --- | --- | --- | --- | --- | --- | --- | --- |
| **fasting glucose** | control | 138 | 127 | 121 | 122 | 114 | 104 | 107 |
|  | basal | 142 | 129 | 120 | 113 | 111 | 103 | 102 |
|  | insulin pump | 128 | 114 | 105 | 101 | 102 | 98 | 96 |
| SD | control | 26 | 27 | 35 | 33 | 34 | 29 | 30 |
|  | basal | 32 | 25 | 25 | 26 | 27 | 23 | 19 |
|  | insulin pump | 46 | 34 | 29 | 24 | 24 | 20 | 15 |
|  |  |  |  |  |  |  |  |  |
| Mean | DAY | **1** | **2** | **3** | **4** | **5** | **6** | **7** |
| **pre-lunch glucose** | control | 152 | 155 | 151 | 154 | 165 | 188 | 165 |
|  | basal | 153 | 149 | 143 | 152 | 143 | 149 | 140 |
|  | insulin pump | 146 | 155 | 152 | 150 | 149 | 137 | 143 |
| SD | control | 33 | 47 | 40 | 51 | 55 | 47 | 47 |
|  | basal | 50 | 33 | 34 | 46 | 31 | 25 | 33 |
|  | insulin pump | 47 | 48 | 47 | 45 | 41 | 33 | 32 |
|  |  |  |  |  |  |  |  |  |
| Mean | DAY | **1** | **2** | **3** | **4** | **5** | **6** | **7** |
| **pre-supper glucose** | control | 153 | 171 | 186 | 173 | 180 | 204 | 177 |
|  | basal | 148 | 171 | 169 | 173 | 171 | 163 | 162 |
|  | insulin pump | 152 | 156 | 157 | 165 | 168 | 151 | 162 |
| SD | control | 60 | 59 | 56 | 56 | 57 | 59 | 56 |
|  | basal | 30 | 30 | 34 | 36 | 34 | 37 | 39 |
|  | insulin pump | 27 | 39 | 36 | 44 | 40 | 34 | 53 |
|  |  |  |  |  |  |  |  |  |
| Mean | DAY | **1** | **2** | **3** | **4** | **5** | **6** | **7** |
| **post-supper glucose** | control | 164 | 157 | 188 | 185 | 165 | 164 | 180 |
|  | basal | 148 | 171 | 169 | 173 | 171 | 163 | 162 |
|  | insulin pump | 154 | 146 | 165 | 142 | 144 | 136 | 116 |
| SD | control | 30 | 23 | 54 | 35 | 47 | 49 | 33 |
|  | basal | 30 | 30 | 34 | 36 | 34 | 37 | 39 |
|  | insulin pump | 45 | 45 | 51 | 40 | 26 | 27 | 26 |
