# Supplementary material for: A randomized controlled trial-based algorithm for insulin-pump therapy in hyperglycemic patients early after kidney transplantation
Source: PLoS One. 2018 Mar 8;13(3):e0193569. doi: 10.1371/journal.pone.0193569 (PMC5843249; doi:10.1371/journal.pone.0193569)

Insulin Therapy for the Prevention of New Onset Diabetes after Transplantation (ITP-NODAT)

Prospective Study in Non-Diabetic De Novo Kidney Transplant Recipients

Protocol authors — *Prof. Dr. Marcus Säemann, Dr. Manfred Hecking, Dr. Johannes Werzowa, Prof. Dr. Akinlolu Ojo, Prof. Dr. Fu Luan, Prof. Dr. Klemens Budde*

Document type	*Clinical study protocol*
Study phase	*II*
Document status	*Version 6.0*
Date	*July 13th 2015*
Number of pages	

Confidentiality Statement

The information contained in this document, especially unpublished data, is the property of the sponsor of this study, Department of Internal Medicine III, Division of Nephrology and Transplantation, Medical University Vienna. It is therefore provided to you in confidence as an investigator, potential investigator, or consultant, for review by you, your staff, and an Independent Ethics Committee or Institutional Review Board. It is understood that this information will not be disclosed to others without written authorization from the Department of Internal Medicine III, Division of Nephrology and Transplantation, Medical University Vienna, except to the extent necessary to obtain informed consent from those persons to whom the study drug may be administered.

SPONSOR, INVESTIGATOR, MONITOR AND SIGNATURES

Clinical investigator (AMG §§ 2a, 35,36)	*Prof. Dr. Marcus Säemann, Department of Internal Medicine III, Division of Nephrology and Dialysis, Medical University of Vienna, Austria*
Sponsor (AMG §§ 2a, 35,36)	*Medical University of Vienna, Spitalgasse 23, 1090 Wien, Austria*
Monitor (AMG §§ 2a, 35,36)	Carlos Rodriguez-Torres, D.M.D, M.B.A.; Clinical Project Management and Monitoring, Untere Hauptstrasse 66ª, 7453 Steinberg, Austria
Clinical Trial Centers	*- Department of Internal Medicine III Division of Nephrology and Dialysis and Department of Surgery, Division of Transplantation, Medical University of Vienna, Austria*<br>*- Department of Nephrology, Medical University of Graz*<br>*- Department of Nephrology, Charité Berlin, Campus Mitte*<br>*- Department of Nephrology, Charité Berlin, Campus Virchow*<br>*- Hospital del Mar, Barcelona, Spain*

Prof. Dr. Marcus Säemann	Signature	Date
Dr. Rodriguez-Torres	Signature	Date
Prof. DDr. Walter Hörl	Signature	Date
	Signature	Date

Table of Contents

1	**BACKGROUND AND RATIONALE ……………………………………….**	7
1.1	BACKGROUND……………………………………………………………….	7
1.2	RATIONALE…………………………………………………………………..	9
2	**RISKS AND BENEFITS…………………………………………………....**	12
2.1	RISKS…………………………………………………………………………	12
2.2	BENEFITS……………………………………………………………………	13
3	**OBJECTIVES……………………………………………………………….**	14
3.1	PRIMARY OBJECTIVE………………………………………………………	14
3.2	SECONDARY OBJECTIVES………………………………………………	14
4	**STUDY DESIGN AND ENDPOINTS……………………………………….**	15
4.1	STUDY DESIGN……………………………………………………………..	15
4.2	ENDPOINTS…………………………………………………………………..	15
4.2.1	Primary Endpoint………………………………………………………	15
4.2.2	Secondary Endpoints…………………………………………………....	15
5	**STUDY ELIGIBILITY CRITERIA………………………………………..**	17
5.1	INCLUSION CRITERIA………………………………………………………	17
5.2	EXCLUSION CRITERIA……………………………………………………..	17
6	**STUDY SETTINGS, PATIENT ENROLLMENT AND RADOMIZATION………………………………………………………........**	18
6.1	STUDY SETTINGS…………………………………………………………..	18
6.2	PATIENT ENROLLMENT……………………………………………………	18
6.3	RANDOMIZATION AND STRATIFICATION………………………………	18
7	**STUDY PROCEDURES, CONCURRENT THERAPIES, TESTS AND FOLLOW-UP, AND STUDY END…………………………………………....**	19
7.1	STUDY PROCEDURES……………………………………………………..	19
7.1.1	Group A (intervention group)…………………………………………..	19

7.1.2	Group B (standard of care group)………………………………………….....	21
7.2	CONCURRENT THERAPIES………………………………………………	21
7.2.1	Immunosuppression……………………………………………………………	21
7.2.2	Condition other than hyperglycemia and/or NODAT………………………..	22
7.3	TESTS AND FOLLOW-UP……………………………………………………...	22
7.4	END OF STUDY………………………………………………………………….	23
7.4.1	Completion of study…………………………………………………………...	23
7.4.2	Study withdrawal……………………………………………………………...	23
8	**DATA COLLECTION, STORAGE, QUALITY CONTROL AND ASSURANCE ………………………………………………………………..**	24
8.1	DATA COLLECTION…………………………………………………………….	24
8.2	DATA STORAGE………………………………………………………………...	24
8.3	PERMITTING TO ACCESS DATA SOURCE………………………………	24
8.4	QUALITY CONTROL AND ASSURANCE………………………………...	24
8.4.1	Data handling……………………………………………………………………	25
8.4.2	Protection of Confidentiality……………………………………………………	25
9	**STATISTICAL CONSIDERATIONS AND ANALYTICAL PLAN…………………………………………………………………………...**	26
9.1	SAMPLE SIZE AND POWER CALCULATION……………………………..	26
9.2	INTERIM ANALYSES……………………………………………………....	27
9.3	INADEGUATE RECRUITMENT AND EXCESSIVE DROPOUT…….	27
10	**SAFETY MONITORING……………………………………………………...**	28
10.1	DEFINITIONS…………………………………………………………………….	28
10.1.1	Adverse Event…………………………………………………………………….	28
10.1.2	Serious Adverse Event………………………………………………………….	28
10.1.3	Unexpected Adverse Event…………………………………………………….	28
10.2	EVENT COLLECTING AND RECORDING PROCEDURES………………	28
10.3	GRADING AND ATTRIBUTION……………………………………………….	29
10.4	REPORTING CRITERIA………………………………………………………..	29

10.5	REPORTING TIMELINE………………………………………………………..	30
10.6	DATA SAFETY MONITORING BOARD………………………………………	30
11	**ETHICAL CONSIDERATION AND COMPLIANCE WITH GOOD CLINICAL PRACTICE ………………………………………………….....**	32
11.1	INFORMED CONSSENT…………………………………………………....	32
11.2	PRIVACY AND CONFIDENTIALITY………………………………………	32
11.3	PUBLICATION POLICY……………………………………………………..	32
12	**REFERENCES………………………………………………………………….**	34
13	**APPENDICES: FIGURES AND TABLES……………………….................**	39

Glossary of Abbreviation

NODAT: new onset diabetes after transplantation

IFG: impaired fasting glucose

IGT: impaired glucose tolerance

IR: insulin resistance

HOMA: homeostasis model assessment

ESRD: end stage renal disease

PHI: protected health information

DCC: data coordinating center

RedCap: research electronic data capture

A1c: hemoglobin A1c

DSMB: data safety monitoring board

AE: adverse event

SAE: serious adverse event

1. BACKGROUND AND RATIONALE

1.1 Background

Abnormal glucose metabolism after kidney transplantation: Abnormal glucose metabolism is common early after kidney transplantation in previously non-diabetic patients and ranges from impaired fasting glycemia and glucose intolerance to full-blown diabetes (1). New onset diabetes after transplantation (NODAT) represents the most serious form of abnormal glucose metabolism occurring at estimated 25% to 30% kidney transplantation with highest risk in the early period following transplantation (2-6). NODAT incurs elevated risks for major cardiovascular events and has a negative impact on patient and kidney graft survival (7-10). In addition to NODAT, impaired fasting glucose (IFG) and impaired glucose tolerance (IGT) are even more common with prevalence reportedly as high as 36% at 10 weeks and 20 to 30 % beyond the first 6-12 months post-transplantation (1, 11-14).

Hyperglycemia occurs very early after kidney transplantation, within the first week, with 45% of patients having impaired fasting glucose (100 to 125 mg/dl) and 87% of patients having evidence of hyperglycemia (bedside glucose ≥200 mg/dl and/or physician–instituted insulin therapy) (1, 3, 15). Furthermore, recent studies have shown that the determination of early morning fasting glucose in kidney transplant patients exhibits a sensitivity that was too low for an accurate assessment of abnormal glucose metabolism post-transplantation and that pre-dinner time glucose measurements detect more patients with glycemia above 200 mg/dl whose morning fasting glycemia was only minimally abnormal (<126 mg/dl) (13, 16). These data imply that the pattern of hyperglycemia in newly transplanted patients is characterized by rising glucose concentrations throughout the day, but relatively low fasting glucose concentrations in the morning, a phenomenon that has been related to the routine post-transplant use of corticosteroids, with their effects on glycemia peaking between lunch and dinnertime when administered in the morning as typically practiced among the transplant communities (16, 17). Early hyperglycemia and its persistence are risk factors for late development of NODAT.

Among many risk factors, surgery-related stress and the initiation of immunosuppressive agents at high doses, particularly corticosteroids and calcineurin inhibitors such as tacrolimus and cyclosporine, play a critical and negative role on glucose metabolism during early post-transplant period (18-20). Corticosteroids and calcineurin inhibitors are cornerstone of immunosuppressive medications currently employed in kidney transplant patients worldwide. In particular, all patients receive corticosteroids at the time of transplant surgery and more than 60 to 70% of them are maintained on corticosteroids for years during the subsequent follow-up (21, 22). On the other hand, up to 95% of all kidney transplant patients in the US receive tacrolimus (82%) or cyclosporine (12%) (22).

Corticosteroids cause dose dependent peripheral insulin resistance and hepatic gluconeogenesis (12, 23, 24). Calcineurin inhibitors, tacrolimus and

cyclosporine A, impair insulin secretion by directly injuring β-cells (5, 25-28). Although both tacrolimus and cyclosporine A are responsible for the increased risk of NODAT, the diabetogenic property of tacrolimus is much higher than that of cyclosporine (2, 5). In fact, the reported 6 month incidence of NODAT was close to 21% among tacrolimus treated kidney transplant patients, twice as high as among patients receiving cyclosporine (5). The diabetogenic effects of immunosuppressive medications are time and dose dependent as the exposure of transplant patients to those agents is high in the early period following transplantation during which the incidence of abnormal glucose homeostasis is also highest (29). Finally, the early diagnosed post-transplant hyperglycemia and/or NODAT can improve or reverse as the dose of corticosteroids decreases (12). Thus, the early diagnosis of abnormal glucose metabolism could provide opportunity for therapeutic interventions to prevent the development of full blown NODAT.

Insulin resistance (IR) is a common feature associated with type 2 DM and is frequently present in patients affected with metabolic syndrome (MS), a condition characterized by a cluster of clinical traits that include central obesity, impaired fasting glucose, elevated triglycerides, reduced HDL cholesterol, and hypertension (30-33). IR and MS are associated with increased risk for cardiovascular disease, renal disease and type 2 DM (34-37). MS is present in 20 to 40% non-diabetic kidney transplant patients during the first year after transplantation and in a much higher number during the subsequent years (1, 38, 39). MS is associated with reduced transplant renal function and transplant longevity, increased risk for IGT and NODAT, impaired cardiovascular risk profiles and patient death (1, 38, 40-43).

Management of post-transplant hyperglycemia and NODAT: The role of insulin therapy in management of type 2 diabetes mellitus has been confirmed though not always used as the first line of agents, particularly in the early period after the diagnosis has been made. The current guideline in general population recommends the initiation of oral hypoglycemic agents combined with life-style modification while reserving the insulin therapy for those who fail to meet the target goal (44). In recent years, however, the need of aggressively lowering glycemia with the early institution of insulin therapy has been gaining more attention in the management of type 2 diabetic patients among general population (45). As UK Prospective Diabetes Study (UKPDS) has demonstrated the fact that β-cell function progressively deteriorates over time in people with type 2 diabetes mellitus, irrespective of life-style modification and the use of current pharmacological interventions, the concept of β-cell preservation or rejuvenation by aggressively lowering glycemia during early period following the diagnosis of type 2 diabetes mellitus has gained interest and been tested in several clinical studies (46-51). In particular, one recent study involving 382 Chinese patients with newly diagnosed type 2 diabetes mellitus showed that early intensive insulin therapy led to a significantly higher rate of remission at 1 year compared to oral hypoglycemic agents which was accompanied by an improvement in β-cell function (51).

The current approaches in the management of NODAT mirror that of type 2 diabetes mellitus in the general population (19, 52). The importance of early diagnosis for NODAT has been widely recognized (3, 10). However, the importance of early recognition of hyperglycemia below the level for a diagnosis of NODAT, particularly at the time of transplantation while patients are still in the hospital, remains mostly speculative as its long term impact on clinical outcome besides the risk for NODAT is largely unknown (3, 53). Furthermore, studies have shown that early diagnosed NODAT and/or hyperglycemia can improve over time as the exposure to immunosuppressive medications decreases (3, 12). All available data are consistent with the hypothesis that early post-transplant hyperglycemia is largely determined by increased insulin resistance related to the high amount of corticosteroids that more than 60 to 70% of kidney transplant patients receive nationwide, contributed in various degree by decreased insulin production (synthesis and/or release) with the use of calcineurin inhibitors (25, 54-56).

1.2 Rationale

The scientific basis of the current clinical trial is the emerging body of evidence that pancreatic β-cell function can be preserved or rejuvenated if exogenous insulin is used early after the diagnosis of type 2 DM (46-51). The UK Prospective Diabetes Study (UKPDS) demonstrated that β-cell function progressively deteriorates over time in people with type 2 DM notwithstanding life-style modification and the use of step-up care. In parallel, several clinical studies support the notion that β-cell preservation or rejuvenation can be accomplished by controlling hyperglycemia with injectable insulin during the early period following the diagnosis of type 2 DM (46-51). The most compelling evidence in support of this concept is a recent study of 382 Chinese patients with newly diagnosed type 2 DM which showed that early intensive insulin therapy (either as continuous infusion using an insulin pump or as intermittent injection using a combination of long and short acting insulin) for approximately two weeks after the diagnosis of type 2 DM was associated with remission rates after one year that were significantly higher in the insulin groups (51.1% and 44.9%, respectively) compared to a parallel group treated with oral hypoglycemic agents (26.7%). Aside from a type 2 DM risk reduction of 40% to 47% (p=0.0012), β-cell function measured by HOMA B and acute insulin response improved significantly in the early insulin treatment group (51). Aggressive lowering of hyperglycemia with the early institution of insulin therapy in type 2 DM is increasing in clinical practice as well (56).

NODAT differs from type 2 DM of general population in two aspects. First, the time of onset of NODAT can be established with a reasonable precision among transplant patients, whereas the recognition of type 2 DM in general population is often delayed by years. Newly diagnosed type 2 DM in general does not equal newly developed type 2 DM. Second, in addition to some shared risk factors such as old age, obesity and family history, etc., NODAT has a unique set of risk factors, namely immunosuppressive medications, that varies in the intensity of

exposure over the time. These two unique characteristics of NODAT provide the opportunity for preventive intervention.

Non-diabetic end stage renal disease (ESRD) patient population undergoing kidney transplantation represents a unique patient population in which the hypothesis of a beneficial effect of β-cell rest by prompt institution of insulin therapy in patients with exhibited abnormal glucose metabolism when their β-cells are under the maximal stress, surgical and pharmacological, can be tested. Because of the nature of transplantation, the peak of stress is highest at the time of transplantation and shortly afterwards. Therefore, protecting β-cell by aggressively lowering early post-transplant hyperglycemia with the use of insulin therapy may translate into preservation of β-cell function for prolong period of time after kidney transplantation. This approach, in combination with dietary precaution and increased physical activity, may reduce the incidence of NODAT during subsequent months or years.

At this time, the “step-up” strategies are generally recommended for the management of NODAT which involve non-pharmacological therapy, namely life-style modification and dietary precaution, followed by oral hypoglycemic agents, and finally insulin therapy if the target goal for glycemic control is not achieved (57). In addition, there is no established consensus on the management of hyperglycemia that occurs in great majority of non-diabetic kidney transplant patients during early post-transplant period. While life-style modification is an important aspect in the management of NODAT, its’ implementation early after transplantation is much less likely to be successful due to limitation related to recent surgery and high amount of immunosuppressive medications. The resistance to early use of insulin therapy in the management of newly diagnosed type 2 diabetes mellitus in general population to a large extent is related to patients’ desire of avoiding insulin injection that would require increased self-care efforts and fear of potential adverse effects associated with insulin therapy such hypoglycemia and weight gain (58-61). There is no reason to think that kidney transplant patients will behave differently.

Our approach will represent a radical shift from the current paradigm in the management of NODAT which is the only tip of an iceberg of a much larger problem, namely abnormal glucose metabolism. The optimal management of early abnormal glucose metabolism, including screening, diagnosis and treatment, is currently unknown and often depends on the timing after transplant and available resource (14). Complicating the matter further is the dependence of transplant recipients on various immunosuppressive medications with different risk for the emergence of abnormal glucose metabolism and immunological complication of a kidney transplant recipient can have, such as acute rejection requiring therapeutic intervention, particularly early after transplantation (5, 28, 62).

The uniqueness of current study includes emphasis on the improvement of glycemia control immediately after kidney transplantation when, as consequence of surgery and initiation of high doses of immunosuppressive medications, the stress on β-cells is high. Such therapeutic approach allows us to test the

hypothesis that aggressive glucose control with the use of insulin therapy in patients with newly onset post-transplant hyperglycemia, at the time of maximal β-cell stress, will result in reduced incidence of NODAT during subsequent follow-up. Reduction in the incidence of NODAT could have long lasting beneficial effects on cardiovascular risk profile of kidney transplant patients with potential for improvement in kidney graft and patient survival. It could ultimately prove to be cost-effective.

2. RISKS AND BENEFITS

2.1 Risks

Hypoglycemia as a result of insulin therapy: Hypoglycemia resulting from the insulin treatment is the principal and most serious risk associated with this study. However, in comparison to short-acting insulin, episodes of hypoglycemia are reduced with long-acting insulin preparations such as NPH insulin. The most common symptoms or signs of low blood glucose levels (<60 mg/dl) include sweating, lightheadedness/dizziness/fainting spell, palpitation, tremor, hunger sensation, drowsiness, tingling sensation of extremities and lips/mouth, blurred vision, etc. all the way leading to extreme situations such as seizure, unconsciousness/death. The risk factors for developing hypoglycemia include missing or delaying meal, inadvertent insulin overdosing, changing routine physical activities, inter-current illness such as infections or gastroenteritis causing diarrhea and vomiting, drug-drug interaction, sudden declining in transplant renal function, etc. During the period of insulin therapy, patients will be instructed to recognise symptoms indicating possible hypoglycaemia and to obtain immediate capillary blood glucose-levels. In the case of hypoglycemia documented by glucose levels <60 mg/dl or requiring assistance insulin dose will be reduced and/or dose titration will be stopped for 4 days. When mean glucose values in the 120–140 mg/dl range will be obtained, investigators will be allowed to stop titration or temporarily reduce dosage when they believe that further titration will be hazardous. A telephone hotline with study coordinators/investigators on duty 24 hours x7 days per week will be given to all participants for the reporting of hypoglycemia and as needed assistance in such situation.

Inconvenience with daily capillary glucose monitoring and oral glucose tolerance testing: The need for capillary blood glucose monitoring during inpatient and outpatient insulin treatment can be uncomfortable. Appropriated teaching for such monitoring will be provided. The oral glucose tolerance tests require ingestion of 75 grams of glucose dissolved in 250 ml water and additional blood draw. Some subjects may experience some nausea with drinking such concentrated sugar-water. Venipuncture could cause some minor discomfort. Experienced phlebotomists at all academic centers will be employed and the amount of venous blood will be kept at minimum necessary (5 to 7 ml of whole blood, one tsp, at each time).

Weight gain: Weight gain can be attributed to insulin therapy. However, weight gain post-transplantation is a frequent phenomenon and likely of multiple etiology. Patients will be provided detailed dietary counselling and life-style modification as appropriated according to the progress in their recovery following the transplant surgery. The weight changes will be recorded during the course of this study.

Unauthorized data release: There is a remote possibility of unauthorized release of protected health information (PHI) data about study participants. Such disclosure would be extremely unlikely to involve a threat to life, health, or safety but psychological and social risks might occur to participants of the study. The confidentiality of study participants will be maintained by assigning each subject an identification number that will be used to identify subjects in any individual tabulation related to the current study. The link between the identification numbers and patient identity will be maintained but stored separately for future researches. Personal information will be stored in a secure environment, and it will be transferred to the data coordinating center (DCC) using appropriate protections such as password protection and encryption. The study data will be maintained in a secure computer system with standard password protection accessible only to the investigators and dedicated study coordinator. The password will be updated on a quarterly basis. The leftover biological samples will be stored for future researches. Similarly, there is also a remote possibility of inappropriate sample handling. Precaution will be taken to prevent such improper handling: the research freezers are institutionally owned and securely locked with limited access to investigators and study coordinator, all samples are code identified and the link between code and patient's identification will be maintained but stored separately in secured systems.

2.2 Benefits

To the study participants: According to the study primary hypothesis that institution of insulin therapy among de novo previously non-diabetic kidney transplant recipients experiencing early hyperglycemia will result in less new onset diabetes at 1 year after transplantation, the likely benefit among patients assigned to the intervention group will be an overall improved glucose metabolism during subsequent follow-up: normal fasting glycemia, normal glucose tolerance and less diabetes. Even for patients assigned to the control group, being involved in the clinical trial with all the attention paid by investigators and/or study coordinators, they may learn to better handle the potential risks of developing abnormal glucose metabolism post-transplantation.

To the society: On the other hand, the positive outcome of this study will have profound impact on our current understanding of new onset diabetes in patient population as unique as solid organ transplant recipients (kidney, liver, heart etc.) and will change the current paradigm in the management of NODAT. Improvement in glucose metabolism and reduced incidence in NODAT will translate into improved patient and transplant organ survival and ultimately cost-saving for the society.

3. STUDY OBJECTIVES

This study aims to assess the effects of early insulin therapy in previously non-diabetic de novo kidney transplant patients in reducing the incidence of new onset diabetes in particular and abnormal glucose metabolism in general during subsequent follow-up.

3.1 Primary Objective

To determine the clinical efficacy of early initiation of insulin therapy in decreasing the incidence of NODAT among *de novo* kidney transplant patients with manifested post-transplant hyperglycemia early after transplantation.

3.2 Secondary Objectives

1. To determine the improvement in overall glycemic control among patients managed with the early initiation of insulin therapy compared to standard of care management in patients experiencing abnormal glucose metabolism.
2. To determine the improvement in β-cell function among patients assigned to the early initiation of insulin therapy compared to the standard of care management in patients experiencing abnormal glucose metabolism.
3. To determine the effect of early initiation of insulin therapy compared to the standard of care management on quality of life measures.

4. STUDY DESIGN AND ENDPOINTS

4.1 Study Design

This is a prospective randomized multicenter clinical trial with un-blinded endpoint evaluation. There will be two study groups and participants will be enrolled from the University of Michigan and the Medical University of Vienna, Austria (MUV). The MUV study population will include participants from six additional clinical centers in Spain, France, and Germany. The study is an interventional trial comparing aggressive glycemic control with early institution of insulin therapy to standard of care (dietary precaution, life-style modification, oral hypoglycemic agents and/or insulin as needed) (Appendices, figure 1).

This study will involve previously non-diabetic ESRD patients undergoing kidney transplantation with either a deceased or living donor kidney who will receive standard triple immunosuppression regimen including a calcineurin inhibitor (once-daily tacrolimus in Europe, twice-daily tacrolimus in the U.S.), an anti-metabolite (mycophenolate mofetil) and corticosteroids (prednisone) and be followed at each transplant center's outpatient clinic for at least 2 years following transplantation according to the established standard center protocol.

The study plans to enroll 380 patients:

52 patients at the University of Michigan Health System (UMHS) Transplant Center and 328 patients at five European Transplant Centers:

1) Medical University of Vienna, Austria (68 patients)

2) Universitat Autonoma de Barcelona, Spain (90 ± 10 patients)

3) Charité Universitätsmedizin, Campus Charité Mitte, Berlin, Germany (130 ± 10 patients)

4) Medical University of Graz, Austria (40 patients) coordinated by the Medical University Hospital of Vienna, Austria, over a period of 3 years with a minimum of 2 years follow-up.

4.2 Study Endpoints

4.2.1 Primary Endpoint

The incidence of NODAT 12 months after kidney transplantation defined according to American Diabetes Association criteria (63, 64).

4.2.2 Secondary Endpoints

1. The incidence of NODAT at 24 months after kidney transplantation.
2. Glycemia profile during the time of insulin therapy in arm A (intervention) comparing that of arm B (control).
3. The glycemia control using A1c levels, overall and among patients with NODAT, through study period 6, 12 and 24 months after kidney transplantation.
4. Incidence of impaired fasting glycemia and impaired glucose tolerance 6, 12 and 24 months after transplantation.

5. Pancreatic β-cell function at 6, 12 and 24 months after kidney transplantation, measured as insulin secretion during an OGTT in relation to the glucose stimulation (insulinogenic index – total and early phase) (65).
6. Fasting insulin resistance (mostly liver) at 6, 12 and 24 months after kidney transplantation, measured by HOMA-R and by QUICKI (insulin sensitivity) from fasting (basal) glucose and insulin concentration (66, 67).
7. Dynamic insulin sensitivity (mostly muscle and adipose tissues) at 6, 12 and 24 months after kidney transplantation, measured by OGIS and ISIcomp from OGTT data (68).
8. Renal function at 6, 12 and 24 months after kidney transplantation, measured by serum creatinine.
9. Patient and graft survival 6, 12 and 24 months after kidney transplantation.
10. Mental component summary (MCS) and physical component summary (PCS) derived from the Kidney Disease Quality of Life Short Form (KDQoL-SF™) at 6, 12 and 24 months after kidney transplantation.

5. STUDY ELIGIBILITY CRITERIA

5.1 Inclusion Criteria

1. Adult patients with end stage renal disease undergoing kidney transplantation with a deceased or living donor kidney.
2. Absence of diabetes prior to kidney transplantation, defined according to American Diabetes Association guideline (not on oral hypoglycemic agents or insulin with fasting glucose < 126 mg/dl).
3. Receiving standard triple immunosuppressive medications that include tacrolimus (once-daily in Europe, twice-daily in the U.S.), mycophenolate mofetil or mycophenolic sodium and steroids.
4. Capable of understanding the study and willing to give informed written consent for study participation.

5.2 Exclusion Criteria

1. Patients with a diagnosis of diabetes mellitus prior to kidney transplantation, or receiving anti-diabetic medications, or having pre-transplant fasting glucose level equal or greater than 126 mg/dl on two occasions at least three days apart.
2. Patients receiving an organ transplant other than kidney.
3. Patients receiving an unlicensed drug or therapy within one month prior to study entry.
4. Patients with history of hypersensitivity to injectable insulin.
5. Patients with documented HIV infection.

6. STUDY SETTINGS, PATIENT ENROLLMENT AND RANDOMIZATION

6.1 Study Settings

The University of Michigan Transplant Center and the Medical University of Vienna Transplant Center are two principal participating centers in this study. Four additional transplant programs will participate in the study under the coordination of the Medical University of Vienna Transplant Center.

6.2 Patient Enrollment

All patients with end stage renal disease who have no history of diabetes and will undergo kidney transplantation from either a deceased or living donor at the participating transplant centers will be potential study participants.

Potential study participants will be identified through the UMHS Organ Transplantation Information System (Otis) and the Medical University of Vienna Transplant Registry/the Transplant Registry at each participating center in the EU by the time of notification for transplantation. At the time of recruitment, a sincere discussion of the study procedures, risks and benefits, will take place between the dedicated study coordinators/investigators and the prospective study participants. A written informed consent for participation in the entire study will be obtained once patients agree to be part of the proposed study. Confidentiality and protected health information (PHI) will be protected according the institutional regulation. Participation into the study is completely voluntary. Any patient who refuses to take part in the proposed study will continue to receive standard of care at the UMHS transplant center and the respective European transplant programs.

Upon inclusion, study participants will have baseline assessment comprising of comprehensive clinical assessment and laboratory testing.

6.3 Randomization and Stratification

Prior to transplantation, participants will be randomized into one of two study arms, A (intervention arm) and B (standard of care arm). A blocked randomization ratio of 1:1 will be performed at each transplant center. The only criteria for stratification will the number of previous transplant: first transplant versus repeat transplant. A unique randomization number will be generated by a randomization software and is assigned to each patient (patient randomization number). Sealed envelopes will be used for each participant/randomization number. In the envelopes there will be a note stating whether the patient receives insulin therapy or not. The envelopes will be provided by a person not involved in the study. For participants who replace discontinuing participants before they had a drug intake on day 1 of the first investigation period, the substitute participant will receive the same randomization number as the subject he (she) replaces.

7. STUDY PROCEDURES, CONCURRENT THERAPIES, TESTS AND FOLLOW-UP, AND STUDY END

7.1 Study Procedures

7.1.1 <u>Group A (intervention group)</u>

Capillary glucose monitoring will be instituted immediately after the transplant surgery for all participants randomized to the intervention arm: before breakfast (7:30 am), before lunch (12:00 pm), before dinner (5:30 pm) and at bedtime (10:00 pm). This monitoring schedule will be continued during the initial hospitalization for the transplant surgery and after the discharge for a total duration of 4 weeks or longer if insulin therapy is instituted during the period of time (see below). The study investigators and coordinators will make daily assessment of study participants while inpatients with regard to their capillary glucose monitoring values. During the initial hospitalization, participants will receive individualized meal plans that incorporate consistent quantities of carbohydrates at each meal and snack. Carbohydrate content of meals and snacks may vary from individual to individual according to personal preferences and habits, but should be consistent from day to day for each individual. Study participants will also receive appropriate education regarding dietary variation (such as skipping meals or frequent snacks), the capillary glucose monitoring techniques, maintenance of a log recording the 4 times daily capillary glucose values, injection of insulin if insulin therapy is initiated in hospital, recognition of symptoms suggestive of hypoglycemia and its appropriate management, etc. Following discharge, patients will continue 4 times daily capillary glucose monitoring at home and will maintain a log recording deviations from normal diet or physical activity, capillary glucose values and insulin doses. They will be given a toll-free phone number to communicate with study investigators and coordinators for all the necessary assistance in the management of glycemia and other medical care.

During the initial 4 weeks of capillary glucose monitoring, if patients have pre-dinner glucose levels of 140 mg/dl or greater, they will be started on insulin therapy with intermediate acting NPH insulin (human insulin isophane, Humulin N, Eli Lilly) in the morning before breakfast. The initial doses of NPH insulin are as following: 10 units before breakfast for pre-dinner capillary glucose levels between 140 and 179 mg/dl, 12 units between 180 and 239 mg/dl, and 14 units for 240 mg/dl and higher (Appendices, table 1). Once started on insulin therapy, 4 times daily capillary glucose monitoring will be continued. During therapy, the pre-dinner capillary glucose target is 110 mg/dl.

During the inpatient stay following the surgery, the NPH insulin dose titration will be performed in a stepwise fashion according to pre-dinner capillary blood glucose levels. For values that are out of the target range without a clear-cut explanation (such as lunch time hypoglycemia or an unscheduled late afternoon snack): 2 to 4 unit increases or 4 unit decreases over the initial dosage will be made for above or below the target, respectively (Appendices, table 1). The NPH insulin dose adjustment will be made every day for up-titration in response to

otherwise unexplained elevation of pre-dinner glucose and daily for down-titration in response to pre-dinner hypoglycemia. Down-titration may also be performed to account for the anticipated impact of the protocol-driven prednisone taper on glucose metabolism.

Mealtime short acting insulin (Insulin lispro, Humalog, Eli Lilly) will be used to correct pre-lunch and bedtime hyperglycemia only (Appendices, table 2). If pre-lunch glucose levels are persistently elevated, lispro will be added before breakfast the following day. Similarly if bedtime glucose levels are persistently elevated, lispro will be added before dinner meal the following day. The dose selection will be determined from Appendices table 2. For elevation in pre-dinner glucose levels, the adjustment in next morning NPH dose will performed as stated above following the titration scale (Appendices, table 1). If pre-breakfast glucose levels remains consistently above 200 mg/dl, an additional dose of NPH insulin at dinner will be considered at the discretion of the study investigators.

In our previous proof-of-concept clinical trial of basal insulin for post-transplant hyperglycemia at the Medical University of Vienna (Hecking et al., JASN 2012), 2 of 25 patients in the insulin intervention group could not be managed with once-daily isophane insulin and short acting insulin alone, because of severe hyperglycemia. These patients were put on a 70/30 formulation of isophane insulin and short acting insulin up to three times daily. It is currently not possible to determine a specific algorithm for such a treatment regimen in the present intervention group. Therefore, for patients requiring excessive NPH doses (usually ≥30 IU), a 75/25 formulation of intermediate and short acting insulin (Humalog Mix 75/25, Eli Lilly) may be used up to three times daily before breakfast, lunch, and dinner at the discretion of the primary study investigators. According to our previous experience, approximately 65% of the previous total daily insulin (NPH and short acting) should be administered by 2 pm, further dose intensification will be made individually, at the discretion of the primary study investigator, and not according to a pre-specified algorithm.

Following discharge from the initial hospitalization, patients will be contacted daily by the study coordinator/investigator. The insulin dose will be adjusted or insulin will be started according to the protocol (Appendices, table 1). They will be seen in the outpatient clinic on a weekly or biweekly base. Patients will bring their meters and the log of home capillary glucose monitoring results, insulin doses and unusual dietary changes to the clinic for investigators and coordinators to review. The dose of insulin, if started as an inpatient or following discharge but prior to the first clinic visit, will be further adjusted based on capillary glucose values reported during the week or days prior to clinic visit. Additional dose adjustment will be made as appropriate over the phone between clinic visits. Once the target levels of pre-dinner glycemia (≤110 mg/dl) are achieved, patients will continue insulin therapy for 14 additional days with appropriate dose adjustment following the same principle as stated above (dose increases or dose decreases daily for pre-dinner capillary glucose value above or below the target ranges, respectively). After that, a trial of weaning insulin will begin in a stepwise fashion. During the weaning process, the same pre-dinner

glycemia targets will be used. Patients who are weaned off insulin therapy successfully will be monitored for the development of NODAT according to the study protocol during the first two years of kidney transplantation and cared as per the current transplant center practice. For those patients who cannot be successfully weaned off NPH insulin during the first six months post-transplant, NPH insulin treatment will be continued until the study investigators make additional assessment for the appropriateness of further attempts to wean the patient off insulin.

For all patients who do not meet the criteria for the initiation of insulin therapy during the first 4 weeks post-transplant surgery, capillary glucose monitoring will be stopped after the initial 4 weeks.

7.1.2 Group B (standard of care group)

Patients will be managed according to the current transplant centers practice with no routine glycemia monitoring other than once daily fasting glucose level in the morning as part of a basic metabolic panel to follow renal function improvement following the surgery. Patients whose glucose values are above 200 mg/dl will be monitored subsequently and, if confirmed, covered by short-acting insulin according to a sliding scale during their in-hospital stay (Appendices: table 2). If a permanent antidiabetic medication is necessary, sulfonylureas will be the treatment of choice whenever possible. Upon discharge, only patients who cannot be controlled by sulfonylureas and have to be started on insulin will be instructed to have glycemia monitoring at home (four times a day: before each meal and at bedtime). For all others, no home glycemia monitoring will be instituted and patients will be followed up subsequently in the outpatient clinic. Any additional glucose abnormality observed during regular outpatient follow-up will be managed according to the current transplant center standard of care protocol in a stepwise approach: dietary consultation and attendance of diabetes education class, followed by the initiation of oral hypoglycemia agents, usually from the sulfonylurea class, of choice by a transplant physician caring for the given patient, and/or intermediate and long-acting insulin added on as the last step by individual transplant physicians in consultation with an endocrinologist/diabetes specialist of choice.

The data from control patients followed at the Medical University of Vienna will be used as control arm in a related study entitled “Treat-To-Target Trial of Continuous, Sensor-Augmented Insulin-Pump Therapy in New-onset Diabetes after Transplantation (SAPT-NODAT): Efficacy and Safety of an Intensive Insulin Protocol in Renal Transplant Recipients Receiving a Tacrolimus-based Immunosuppression”.

7.2 Concurrent Therapies

7.2.1 Immunosuppression

1. Induction: will be used with approved various induction agents according to center protocol. However, no experimental agents should be used.

2. Maintenance: will consist in once-daily tacrolimus (Europe) or twice-daily tacrolimus (U.S.), twice-daily mycophenolate mofetil and once daily prednisone. The dose of tacrolimus will be adjusted according to 12 hours trough concentration levels as described in the table 3 (Appendices). The dose of mycophenolate will be 2 grams twice daily or according to center practice protocol, and adjusted per adverse effects (gastroenteric intolerance and/or infection, etc.). The dose of prednisone will be tapered uniformly across the study patients as described (Appendices, table 3).

7.2.2 <u>Conditions other than hyperglycemia and/or NODAT</u>

Hypertension, dyslipidemia and other medical conditions will be treated according to the UMHS transplant center's cardiovascular disease prevention and treatment protocols, respectively according to best clinical practice at the European transplant centers, and determined by individual transplant physicians and surgeons caring for the given patients.

Transplant organ function or dysfunction will be assessed according to the current transplant center protocols by individual transplant physicians and surgeons caring for the given patients. Appropriate diagnostic tests and/or procedures including transplant kidney biopsy will be ordered regardless their status as a participant of the study or whether they develop NODAT or not.

Requirement for hospital admission for the condition other than related to the study will be determined by individual transplant physicians and surgeons. Once in the hospital, 4 times daily capillary glucose monitoring will be instituted for study participants from group A. Study investigators and coordinator will make daily assessment of their glycemic control and/or make appropriate insulin dose adjustment during their inpatient stay.

7.3 Tests, Procedures and Follow-up

At the time of enrollment, fasting glucose and insulin levels (at least 8 hours from the previous meal) and A1c levels will be obtained. An additional whole blood sample for potential future genetic studies related to diabetes risk, and leftover biological samples (serum and urine) will be obtained with study participant's informed consent. However, this remains optional. A refusal for such consent does not disqualify patients from the study. Once discharged from the initial transplant hospitalization, for study participants randomized to the interventional arm (group A), the study coordinator/investigator will contact them daily inquiring the glucose monitoring results from the previous day whether they are started or not on insulin therapy during the initial hospitalization and guide them through the initiation and/or titration of insulin according the protocol (see above 7.1.1). All study patients will have study visits (coinciding with their routine transplant care visits whenever possible) in the transplant center outpatient clinic weekly for the first month, and biweekly in the second and third months. Subsequently, patients will be seen every three months during first post-transplant year and every 4 months during second post-transplant year (Appendices, table 4). During the study visits, investigators and coordinators will review and record the log sheet of home glucose monitoring, insulin dosage (NPH and/or short-acting

coverage) change, diary of dietary changes/additional snacks, occurrence of adverse reaction (episodes of hypoglycemia, etc.) and perform study related procedures at pre-determined time points (Appendices, table 4). In particular, 2 hours oral glucose tolerance test will be performed at 6, 12 and 24 months (the results of 6 months OGTT will be blinded to study participants and investigators/coordinators). Their assigned transplant physicians will be in charge for their overall post-transplant care. Study related biological sample collection includes blood and urine samples as outlined in table 4. Unless otherwise specified (2 hours post glucose challenge) all blood samples will need to be collected at fasting state (at least 8 hours from the previous meals).

Study related OGTT will be performed in the outpatient clinical research unit. On the day of testing, the study participant will arrive in the morning in a fasting state (at least 8 hours from the previous meal). An angiocatheter will be placed in a peripheral vein of forearm. A first blood sample will be collected (time 0, fasting). Thereafter, the study participant will ingest 75 grams of glucose dissolved in water. Subsequent blood samples will be collected at 30, 60, 90 and 120 minutes. The plasma will be separated promptly for the whole blood and stored at - 80°C for various study-related measurements in a late time.

7.4 End of study

7.4.1 <u>Completion of study</u>

The completion of study is 24 months after the kidney transplantation. An end of study visit will be scheduled within a 2-week time frame with appropriate study related tests and/or procedure. Patients will continue to receive care for their medical conditions related or not to the study as per the UMHS transplant center, respectively the participating European transplant centers’ standard of care.

7.4.2 <u>Study withdrawal</u>

Patients may discontinue prematurely their participation in the study anytime for any of the following reasons:

1. At their own request.
2. Investigators feel that continuation in the study is not at the best interest of patients.
3. Patient physicians feel that continuation in the study is not at the best interest of patients.
4. DSMB feels that continuation in the study is not at the best interest of patients.

A visit for the end of study will be scheduled within 10 days of premature withdrawal and the reasons of withdrawal will be documented. Patients will continue to receive care for their medical conditions according to the current UMHS transplant center, respectively the participating European transplant centers’ protocols. No further study related tests and/or procedure will be performed. All study materials up to the time of withdrawal will be retained and used for the final analyses unless a specific request is made otherwise.

8. DATA COLLECTION, STORAGE, QUALITY CONTROL AND ASSURANCE

8.1 Data Collection

The proposed study will use paper acse report forms.

In addition, data may be entered into a web-distributed data entry and a fully relational clinical research data management on the *Research Electronic Data Capture (RedCap)*. *RedCap* is a commercially-developed regulatory-compliant web-based clinical research information system licensed to and supported by the Michigan Institute for Clinical and Health Research (MICHR) and CTSA Biomedical Informatics Program (BIP). *RedCap* will be used for protocol management, subject scheduling and tracking, data management, reporting, budget and milestones, and account management. The study will also use *RedCap* for integrated specimen management, and integration/federation of data with other research information systems, research administrative systems (IRB, consent management, research billing), and molecular databases. All Case Report Forms (CRF) and questionnaires will be deployed in *RedCap*. Study personnel will undergo a central training in the use of *RedCap* which will be supplemented with annual recertification. *RedCap* will be used to generate quality control reports. Edit checks will be performed online as the data is entered so that laboratory results can be seamlessly loaded into the database. Data collection forms and data entry screens will be aesthetically pleasing with minimal graphics to facilitate ease of use and to minimize keyboard response time. The questions on the data forms will be constructed in easy to understand language and placed in a logical sequence. Edit checking will be conducted for codes, dates and ranges on an ongoing basis. A reliable rekey verification system (twice entered) will be utilized for critical data elements. A keystroke error rate in excess of 0.15% will trigger inquiry, retraining or other corrective steps that may be indicated. *RedCap* meets 21 CFR part 11 and HIPAA regulatory standards, has built-in security features including login/password access, data authentication, user-based access to subject information, code-base identification, ability to suppress subject information and automatic log-off. In addition, *RedCap* uses Secure Sockets Layer (SSL) encryption software for online protection and has protection capabilities for off-line use as well.

8.2 Data Storage

Data will be stored at each participating center in paper form. The University of Michigan Transplant Center will serve as overall Data Coordinator Center (DCC) whereas Medical University of Vienna will serve as European data collection center for data generated through various participant European study centers. Final data will converge to the University of Michigan Transplant Center after full financial recompensation of the European centers.

8.3 Permitting Access to Source Data

Individual centers will be allowed to access the data of their own study participants for the purpose of quality control and assurance,

8.4 Quality Control and Assurance

The DCC will be responsible for regularly reviewing the conduct of the data collection process, for verifying adherence to the protocol, and for confirming the completeness, consistency, and accuracy of all documented data.

8.4.1 Data Handling

The investigator is required to ensure that all CRFs are completed for every participant entered in the trial. The data will be verified by a series of computerized edit checks, and all relevant data queries will be resolved regularly. When the CRFs are complete, they will be reviewed and signed by the investigator. All data from the original signed CRF will be then entered in the database, and a comparison program will be run again. All discrepancies will be reviewed, and any resulting queries will be resolved with the investigator and amended in the database. All elements of data entry (i.e., time, date, verbatim text, and the name of the person performing the data entry) will be recorded in an electronic audit trail to allow all data changes in the database to be monitored and maintained in accordance with federal regulations.

8.4.2 Protection of Confidentiality

Researchers will electronically transfer the source data to the DCC (the European centers to the MUV). The DCC will collect, update and verify data on the study variables from the following sources at the respective transplant programs: archived paper medical records; system-wide and program-specific clinical information data management systems; and their respective organ transplant information systems.

9 STATISTICAL CONSIDERATION AND ANALYSTIC PLAN

9.1 Sample Size and Power Calculations

The sample size and power calculations revealed that 276 participants will be needed to have 85% power to detect a 15% difference in the proportions of participants who develop NODAT under the assumption that the baseline incidence of NODAT in the control treatment (group B) will be 30% at 12 months (1, 4). We plan to enroll at least 300 participants with a constant accrual rate. We estimate that the rate of loss to follow-up will be 6% in year one and 8% in year two (Appendices, table 5). These attrition rates would leave an end-of-study sample size of 280 participants out of the 300 enrolled. For the secondary endpoint, sample size calculations revealed that 54 participants (Appendices, table 6) will be needed to have 85% power to detect a difference of 0.5% in hemoglobin A1c under the assumption that the mean hemoglobin A1c at 12 months post-transplantation will be 6.3% in participants randomized to the control treatment (group B). The power calculations are based on two-sided test of significance with an alpha level of 0.05.

The two study groups will be compared using the Student t-test for continuous variables and the χ^2 or Fisher's exact test for categorical variables. We will evaluate the effect of the early insulin regimen *vs.* step-up approach on the risk of NODAT using the Cox proportional hazards model for the time to the failure event, adjusted for the clinical center effect (UMHS vs. other transplant centers). The Cox model takes the form λ(t) = λ0(t)expβ1d + β2s + β3(c), where λ(t) is the hazard function at time t and λ0(t) is the baseline hazard function, with treatment *d* (group assignment) and clinical center *s* being covariates of interest as defined. The hazard ratio comparing early insulin regimen to the step-up approach is quantified by eβ1. The null hypothesis is H0: β1=0, that is, the two treatment groups have the same hazard; we wish to show H1: β1 ≠ 0. At the type I error 0.05, the sample size needed with Brian O Fleming adjustment for one interim analysis is shown in table 5. The sample size calculation is based on a 1:1 randomization ratio between the experimental arm (group A) and the control arm (group B). The Cox regression model's assumption of proportionality will be assessed graphically using plots of Schoenfeld residuals against time and numerically via time-dependent interaction terms. Significant violations of the assumption by one or more covariates will be handled using time-dependent interactions or stratification of the model on the covariate. The planned sample size of 300 subjects with annualized rate of loss to follow-up of 6% (year 1) and 8% (year 2) yield a power of 85% to detect a relative reduction of 0.50 (RR = 0.50).

For the secondary endpoint of hemoglobin A1c, we will use the Cochran-Mantel-Haenszel test to assess the relationship between study groups and mean hemoglobin A1c. Based on our preliminary studies, we estimated that the mean hemoglobin A1c in the control treatment group (group 1) would be 6.3 + 0.7. Table 6 shows that the sample size is sufficiently robust to find a 0.5% difference in hemoglobin A1c if a power level of 85% is selected.

9.2 Interim Analyses to Ensure Patient Safety

Interim analyses for therapeutic efficacy will not be required for this study. However, regular monitoring for the occurrence of adverse events will be conducted and assessed among all study participants.

9.3 Inadequate Recruitment and Excessive dropout

The most significant problem for any clinical trial is the ability to meet the recruitment goals within the specified timeline. Our preliminary assessment indicates that we will have more than 700 potentially eligible participants at least, at the University of Michigan and the Medical University of Vienna alone, combined over the 30-month enrolment period. Our experience over the last 20 years suggests that we would achieve a participation rate of 50-60% among eligible participants leaving us with ample pool of potential participants. To ensure that we address recruitment problems in a timely fashion, each of the clinical centers will be required to maintain a screening and recruitment log in *RedCap* and at the clinical center. The recruitment log will also be used to document the magnitude and reasons for non-participation by eligible patients. A color-coded 3-stagewarning system will be utilized to manage the recruitment process on a monthly basis. A recruitment efficiency (R) will be automatically calculated according to the formula R= ∑(tk □ tk□1)[r(tk□1) + r(tk)], where tk is the proportion of the recruitment period by unit of time k, the variable r is the fraction of the planned sample size recruited by time k and the variable k defines the entire recruitment period. The value of r for both Michigan and Vienna will be set at a target of 110%. A drop in the recruitment efficiency below 80% for two consecutive months (orange alert) will trigger an immediate review of all aspects of the recruitment operation by Drs. Ojo and Säemann. In order to protect the power for statistical analyses of main study hypotheses, it would be necessary to maintain a retention rate of 85% by the end of the study. Specific measures that will be taken to enhance retention of participants in the study include: (a) regular contact with referring physician practices; (b) use of only trained, certified study personnel to perform study visits and; (c) face-to-face meeting between study physician and each participant at least quarterly during study visits. Established practices such as mailings and personal telephone calls to remind participants about forthcoming study visits will also be incorporated. The study team will promptly provide results of study tests to referring physicians. Participants will be encouraged to include family members in their follow-up visits since this may enhance the participant support without extra costs. Follow-up visit appointments will be scheduled with large appointment windows to allow for more flexibility and participant’s convenience.

10 SAFETY MONITORING

10.1 Definitions

10.1.1 Adverse Event

An adverse event (AE) is any occurrence or worsening of an undesirable or unintended sign, symptom (including an abnormal laboratory finding), or disease that is temporally associated with the use of a medicinal product whether considered related to the medicinal product or not.

An adverse event will be followed until it is resolved or until 30 days after a participant terminates from the study, whichever comes first.

10.1.2 Serious Adverse Event

A serious adverse event (SAE) is defined as “any adverse event occurring at any dose that suggests a significant hazard, contraindication, side effect, or precaution.” This includes, but is not limited to, any of the following events:

1. Death: A death that occurs during the study or that comes to the attention of the investigator during the protocol-defined follow-up after the completion of therapy must be reported whether it is considered to be treatment related or not.
2. A life-threatening event: A life-threatening event is any adverse therapy experience that, in the view of the investigator, places the patient or participant at immediate risk of death from the reaction as it occurred.
3. Inpatient hospitalization or prolongation of existing hospitalization due to hypoglycemia, as well as hypoglycemias with blood glucose ≤40 mg/dL, shall be judged as SAEs, in analogy to the NICE-SUGAR study (*N Engl J Med.* 2009 Mar 26;360(13):1283-97).
4. Inpatient hospitalization or prolongation of existing hospitalization due to an unexpected cause. Rejection episodes, infections and urological complications requiring hospitalization after transplantation are clearly exempt from this definition, because they are not unexpected after transplantation. Such events are –by definition- not recorded as SAEs.
5. Persistent or significant disability.

Regardless of the relationship of the adverse event to study drug, the event must be reported as an SAE if it meets any of the above definitions.

10.1.3 Unexpected Adverse Event

An adverse event is considered “unexpected” when its nature (specificity) or severity is not consistent with applicable product information, such as safety information provided in the package insert, the investigational plan, or the investigator’s brochure.

10.2 Event Collecting and Recording Procedure

Adverse events will be collected from the time the participant begins study treatment until the time the event is resolved or until 30 days after the participant completes study treatment, whichever comes first.

Adverse events may be discovered through any of these methods:

1. Observing the participant during clinical trial period.
2. Questioning the participant at interval follow-up visits which should be done in an objective manner.
3. Receiving an unsolicited complaint from the participant.
4. An abnormal value or result from a clinical or laboratory evaluation (e.g., a test result, a radiograph, an ultrasound, or an electrocardiogram) can also indicate an adverse event. If this is the case, then the evaluation that produced the value or result should be repeated until the value or result returns to normal or can be explained and the participant's safety is not at risk. If an abnormal value or result is determined by the investigator to be clinically significant, it must be indicated as such on the appropriate laboratory evaluation form(s), and must also be reported as an adverse event on the adverse event form.

Throughout the study the investigator will record all adverse events on the appropriate adverse event case report form (CRF) regardless of their severity or relation to study medication or study procedure. The investigator will treat participants experiencing adverse events appropriately and observe them at suitable intervals until their symptoms resolve or their status stabilizes.

10.3 Grading and Attribution

The study site will grade the severity of adverse events experienced by study participants according to the criteria set forth in the National Cancer Institute's *Common Terminology Criteria for Adverse Events Version 3.0* (NCT-CTCAE published June 10, 2003). This document (referred to herein as the NCI-CTCAE manual) provides a common language to describe levels of severity, to analyze and interpret data, and to articulate the clinical significance of all adverse events.

Adverse events will be graded on a scale from 1 to 5 according to the following standards in the NCI-CTCAE manual:

Grade 1 = Mild adverse event.

Grade 2 = Moderate adverse event.

Grade 3 = Severe and undesirable adverse event.

Grade 4 = Life-threatening or disabling adverse event.

Grade 5 = Death.

All adverse events will be graded whether they are or are not related to disease progression or treatment.

10.4 Reporting Criteria

After the AE and/or SAE have been assessed, the event will be reported to the appropriate health authorities in the required manner based on the following criteria:

1. No reporting: This requirement applies if the adverse event is deemed not serious by the medical liaison, the medical monitor, and the NIAID Medical Officer.
2. Standard reporting: This requirement applies if the adverse event is classified as any of the following:

   a. Serious, expected, and drug related.

   b. Serious, *unexpected,* and not drug related.
3. Expedited reporting: This requirement applies if the adverse event is considered serious, unexpected, and drug related. This type of SAE must be reported by the sponsor to the appropriate health authorities within 15 days; fatal or life-threatening events must be reported within 7 days.

10.5 Reporting Timeline

When an investigator identifies an SAE (as defined in section **10.1.2**), he or she must notify the sponsor (at MUV), the monitor, and the company providing study drug (Eli Lilly) and the local Ethics Committee within 24 hours of discovering the event. In addition to telephone reporting, the investigator must ensure that these events are entered on the SAE report form and the adverse event CRF.

The investigator will ensure the timely dissemination of SAE information, including expedited reports, to the IRB in accordance with applicable regulations and guidelines.

10.6 Data Safety Monitoring Board

A Data and Safety Monitoring Board (DSMB) will convene to act in an advisory capacity to monitor participant safety and evaluate the ability of the investigators to conduct the proposed with utmost regard for participant protection and confidentiality. The DSMB will undertake the following tasks:

1. Approve the initiation of the proposed study.
2. Review the research protocol, informed consent documents, and plans for data safety and monitoring, including all proposed revisions.
3. Evaluate the progress of the study, including periodic assessments of data quality and timeliness, participant recruitment, accrual and retention, participant risk versus benefit, performance of the clinical centers, and other factors that may affect study outcomes.
4. Consider factors external to the study when relevant information becomes available, such as scientific or therapeutic developments that may have an impact on the safety of the participants or the ethics of the study.
5. Protect the safety of the study participants and report on the safety and progress of the study.
6. Make recommendations to the study team and, if required, to the FDA and IRBs concerning continuation, termination, or other modifications of the study based on the observed beneficial or adverse effects of the study procedures.

7. Review interim analysis data in accordance with stopping rules that are defined in advance of data analysis and have the approval of the DSMB.
8. Ensure the confidentiality of the clinical trial data and the results of monitoring and assist the investigators by commenting on any problems with study conduct, enrollment, sample size, and/or data collection.

The DSMB will consist of five members: one biostatistician from the University of Michigan, one nephrologist and one endocrinologist each from the UMHS and MUV. Members of the DSMB shall have no financial, scientific, or other conflict of interest with the study. Collaborators or associates of the investigators in this application would not be eligible to serve on the DSMB. Written documentation attesting to absence of interest will be required of the DSMB members. All aspects of the clinical trial described in this application will be conducted with utmost regard for the welfare and privacy of the volunteer participants. The DSMB shall conduct periodic meetings at a frequency no less than once every 6 months. The DSMB meetings shall be closed to the public because discussions may address confidential participant data. An emergency meeting of the DSMB may be called at any time by the Chair of the DSMB in the event of issues regarding participant safety. The format for DSMB meetings may be open, closed and/or executive session, as dictated by the agenda of the meeting.

In case that the European part of the study has to be separated form the U.S., the European representatives of the DSMB along with the monitor will take over full responsibilities for the European part of the study.

11. ETHICAL CONSIDERATION AND COMPLIANCE WITH GOOD CLINICAL PRACTICE

Before study initiation, the protocol will be reviewed and approved by an appropriate Internal Review Board (IRB) any by the Competent Authorities as applicable per local laws and regulations. Any amendments to the protocol must also be approved before they are implemented.

11.1 Informed Consent

The informed consent form is a means of providing information about the trial to a prospective participant and allows for an informed decision about participation in the study. All participants (or their legally acceptable representative) must read, sign, and date a consent form before entering the study, taking study drug, or undergoing any study-specific procedures. Consent materials for participants who do not speak or read English must be translated into the participants' appropriate language.

The informed consent form must be revised whenever important new safety information is available, whenever the protocol is amended, and/or whenever any new information becomes available that may affect participation in the trial.

A copy of the informed consent will be given to a prospective participant for review. The attending physician, in the presence of a witness, will review the consent and answer questions. The prospective participant will be told that being in the trial is voluntary and that he or she may withdraw from the study at any time, for any reason.

We will obtain informed consent from all study participants before conducting any study-related testing. Since the investigators and study coordinators approaching the study participants may be the same individuals who participated in their care during subsequent follow-up, extra precautions will be taken to ensure that the potential participants are not coerced or perceive coercion to participate in the study.

11.2 Privacy and Confidentiality

Each participant's privacy and confidentiality will be respected throughout the study. Each participant will be assigned a sequential identification number, and these numbers rather than names, will be used in analytic datasets. The study will require use of identifying information during the stages of (1) data collection from the centers, and (2) database linkages.

11.3 Publication Policy

A publication committee will be established among the study PIs. Any abstract and/or manuscript generated by participating investigators using the data from the current study will be submitted to the committee for review. Each member of the subcommittee will have 2 weeks of time to review the documents for submission to a publishing entity (scientific meetings and/or peer reviewed scientific journals). The member can approve the documents by 1) explicit approval or 2) no objection within the time frame of two weeks.

The authorship will be determined by the publication committee at the time of approval for submission.

11 REFERENCES

1. Luan FL, Stuckey LJ, Ojo AO. Abnormal glucose metabolism and metabolic syndrome in non-diabetic kidney transplant recipients early after transplantation. Transplantation2010 Apr 27;89(8):1034-9.
2. Kasiske BL, Snyder JJ, Gilbertson D, Matas AJ. Diabetes mellitus after kidney transplantation in the United States. Am J Transplant2003 Feb;3(2):178-85.
3. Cosio FG, Kudva Y, van der Velde M, et al. New onset hyperglycemia and diabetes are associated with increased cardiovascular risk after kidney transplantation. Kidney Int2005 Jun;67(6):2415-21.
4. Heisel O, Heisel R, Balshaw R, Keown P. New onset diabetes mellitus in patients receiving calcineurin inhibitors: a systematic review and meta-analysis. Am J Transplant2004 Apr;4(4):583-95.
5. Vincenti F, Friman S, Scheuermann E, et al. Results of an international, randomized trial comparing glucose metabolism disorders and outcome with cyclosporine versus tacrolimus. Am J Transplant2007 Jun;7(6):1506-14.
6. Woodward RS, Schnitzler MA, Baty J, et al. Incidence and cost of new onset diabetes mellitus among U.S. wait-listed and transplanted renal allograft recipients. Am J Transplant2003 May;3(5):590-8.
7. Cosio FG, Pesavento TE, Kim S, Osei K, Henry M, Ferguson RM. Patient survival after renal transplantation: IV. Impact of post-transplant diabetes. Kidney Int2002 Oct;62(4):1440-6.
8. Miles AM, Sumrani N, Horowitz R, et al. Diabetes mellitus after renal transplantation: as deleterious as non-transplant-associated diabetes? Transplantation1998 Feb 15;65(3):380-4.
9. Revanur VK, Jardine AG, Kingsmore DB, Jaques BC, Hamilton DH, Jindal RM. Influence of diabetes mellitus on patient and graft survival in recipients of kidney transplantation. Clin Transplant2001 Apr;15(2):89-94.
10. Hjelmesaeth J, Hartmann A, Leivestad T, et al. The impact of early-diagnosed new-onset post-transplantation diabetes mellitus on survival and major cardiac events. Kidney Int2006 Feb;69(3):588-95.
11. Alberti KG, Zimmet PZ. Definition, diagnosis and classification of diabetes mellitus and its complications. Part 1: diagnosis and classification of diabetes mellitus provisional report of a WHO consultation. Diabet Med1998 Jul;15(7):539-53.
12. Hjelmesaeth J, Hartmann A, Kofstad J, Egeland T, Stenstrom J, Fauchald P. Tapering off prednisolone and cyclosporin the first year after renal transplantation: the effect on glucose tolerance. Nephrol Dial Transplant2001 Apr;16(4):829-35.
13. Sharif A, Moore RH, Baboolal K. The use of oral glucose tolerance tests to risk stratify for new-onset diabetes after transplantation: An underdiagnosed phenomenon. Transplantation2006 Dec 27;82(12):1667-72.
14. Armstrong KA, Prins JB, Beller EM, et al. Should an oral glucose tolerance test be performed routinely in all renal transplant recipients? Clin J Am Soc Nephrol2006 Jan;1(1):100-8.
15. Chakkera HA, Weil EJ, Castro J, et al. Hyperglycemia during the immediate period after kidney transplantation. Clin J Am Soc Nephrol2009 Apr;4(4):853-9.

16. Shah A, Kendall G, Demme RA, et al. Home glucometer monitoring markedly improves diagnosis of post renal transplant diabetes mellitus in renal transplant recipients. Transplantation2005 Sep 27;80(6):775-81.
17. Beard JC, Halter JB, Best JD, Pfeifer MA, Porte D, Jr. Dexamethasone-induced insulin resistance enhances B cell responsiveness to glucose level in normal men. Am J Physiol1984 Nov;247(5 Pt 1):E592-6.
18. Hjelmesaeth J, Hartmann A, Kofstad J, et al. Glucose intolerance after renal transplantation depends upon prednisolone dose and recipient age. Transplantation1997 Oct 15;64(7):979-83.
19. Davidson J, Wilkinson A, Dantal J, et al. New-onset diabetes after transplantation: 2003 International consensus guidelines. Proceedings of an international expert panel meeting. Barcelona, Spain, 19 February 2003. Transplantation2003 May 27;75(10 Suppl):SS3-24.
20. van den Berghe G, Wouters P, Weekers F, et al. Intensive insulin therapy in the critically ill patients. N Engl J Med2001 Nov 8;345(19):1359-67.
21. Luan FL, Steffick DE, Gadegbeku C, Norman SP, Wolfe R, Ojo AO. Graft and patient survival in kidney transplant recipients selected for de novo steroid-free maintenance immunosuppression. Am J Transplant2009 Jan;9(1):160-8.
22. US Oragn Procurement and Transplantation Network, the Scientific Registry of Transplant Recipients. 2008 Annual Report, Transplant Data 1994-2007. . In: Department of Health and Human Service, Human Resource and Service Administration, Healthcare System Bureau, Division of Transplantation, United Network for Organ Sharing, Arbor Research Collaborative for Health, editors. Rockville, Richmond, Ann Arbor2008.
23. Christiansen E, Andersen HB, Rasmussen K, et al. Pancreatic beta-cell function and glucose metabolism in human segmental pancreas and kidney transplantation. Am J Physiol1993 Mar;264(3 Pt 1):E441-9.
24. Goldstein RE, Wasserman DH, McGuinness OP, Lacy DB, Cherrington AD, Abumrad NN. Effects of chronic elevation in plasma cortisol on hepatic carbohydrate metabolism. Am J Physiol1993 Jan;264(1 Pt 1):E119-27.
25. Duijnhoven EM, Boots JM, Christiaans MH, Wolffenbuttel BH, Van Hooff JP. Influence of tacrolimus on glucose metabolism before and after renal transplantation: a prospective study. J Am Soc Nephrol2001 Mar;12(3):583-8.
26. Menegazzo LA, Ursich MJ, Fukui RT, et al. Mechanism of the diabetogenic action of cyclosporin A. Horm Metab Res1998 Nov;30(11):663-7.
27. van Hooff JP, Christiaans MH, van Duijnhoven EM. Evaluating mechanisms of post-transplant diabetes mellitus. Nephrol Dial Transplant2004 Dec;19 Suppl 6:vi8-vi12.
28. Luan FL, Zhang H, Schaubel DE, et al. Comparative risk of impaired glucose metabolism associated with cyclosporine versus tacrolimus in the late posttransplant period. Am J Transplant2008 Sep;8(9):1871-7.
29. Hagen M, Hjelmesaeth J, Jenssen T, Morkrid L, Hartmann A. A 6-year prospective study on new onset diabetes mellitus, insulin release and insulin sensitivity in renal transplant recipients. Nephrol Dial Transplant2003 Oct;18(10):2154-9.
30. Reaven GM. Banting lecture 1988. Role of insulin resistance in human disease. Diabetes1988 Dec;37(12):1595-607.

31. Sulanc E, Lane JT, Puumala SE, Groggel GC, Wrenshall LE, Stevens RB. New-onset diabetes after kidney transplantation: an application of 2003 International Guidelines. Transplantation2005 Oct 15;80(7):945-52.
32. Executive Summary of The Third Report of The National Cholesterol Education Program (NCEP) Expert Panel on Detection, Evaluation, And Treatment of High Blood Cholesterol In Adults (Adult Treatment Panel III). Jama2001 May 16;285(19):2486-97.
33. Grundy SM, Cleeman JI, Daniels SR, et al. Diagnosis and management of the metabolic syndrome: an American Heart Association/National Heart, Lung, and Blood Institute Scientific Statement. Circulation2005 Oct 25;112(17):2735-52.
34. Chen J, Muntner P, Hamm LL, et al. Insulin resistance and risk of chronic kidney disease in nondiabetic US adults. J Am Soc Nephrol2003 Feb;14(2):469-77.
35. Facchini FS, Hua N, Abbasi F, Reaven GM. Insulin resistance as a predictor of age-related diseases. J Clin Endocrinol Metab2001 Aug;86(8):3574-8.
36. Espinola-Klein C, Rupprecht HJ, Bickel C, et al. Impact of metabolic syndrome on atherosclerotic burden and cardiovascular prognosis. Am J Cardiol2007 Jun 15;99(12):1623-8.
37. McNeill AM, Rosamond WD, Girman CJ, et al. The metabolic syndrome and 11-year risk of incident cardiovascular disease in the atherosclerosis risk in communities study. Diabetes Care2005 Feb;28(2):385-90.
38. Porrini E, Delgado P, Bigo C, et al. Impact of metabolic syndrome on graft function and survival after cadaveric renal transplantation. Am J Kidney Dis2006 Jul;48(1):134-42.
39. Luan FL, Langewisch, E., Ojo A. Metabolic syndrome and new onset diabetes after transplantation in kidney transplant recipients. Clin Transplant2010;24.
40. Brodov Y, Chouraqui P, Goldenberg I, Boyko V, Mandelzweig L, Behar S. Serum Uric Acid for Risk Stratification of Patients with Coronary Artery Disease. Cardiology2009 Sep 23;114(4):300-5.
41. Fang J, Alderman MH. Serum uric acid and cardiovascular mortality the NHANES I epidemiologic follow-up study, 1971-1992. National Health and Nutrition Examination Survey. JAMA2000 May 10;283(18):2404-10.
42. Ekstrand AV, Eriksson JG, Gronhagen-Riska C, Ahonen PJ, Groop LC. Insulin resistance and insulin deficiency in the pathogenesis of posttransplantation diabetes in man. Transplantation1992 Mar;53(3):563-9.
43. de Vries AP, Bakker SJ, van Son WJ, et al. Insulin resistance as putative cause of chronic renal transplant dysfunction. Am J Kidney Dis2003 Apr;41(4):859-67.
44. Nathan DM, Buse JB, Davidson MB, et al. Management of hyperglycemia in type 2 diabetes: A consensus algorithm for the initiation and adjustment of therapy: a consensus statement from the American Diabetes Association and the European Association for the Study of Diabetes. Diabetes Care2006 Aug;29(8):1963-72.
45. Bloomgarden ZT. Exploring treatment strategies for type 2 diabetes. Diabetes Care2007 Oct;30(10):2737-45.
46. Intensive blood-glucose control with sulphonylureas or insulin compared with conventional treatment and risk of complications in patients with type 2 diabetes (UKPDS 33). UK Prospective Diabetes Study (UKPDS) Group. Lancet1998 Sep 12;352(9131):837-53.

47. Effect of intensive blood-glucose control with metformin on complications in overweight patients with type 2 diabetes (UKPDS 34). UK Prospective Diabetes Study (UKPDS) Group. Lancet1998 Sep 12;352(9131):854-65.
48. Wajchenberg BL. beta-cell failure in diabetes and preservation by clinical treatment. Endocr Rev2007 Apr;28(2):187-218.
49. Ilkova H, Glaser B, Tunckale A, Bagriacik N, Cerasi E. Induction of long-term glycemic control in newly diagnosed type 2 diabetic patients by transient intensive insulin treatment. Diabetes Care1997 Sep;20(9):1353-6.
50. Ryan EA, Imes S, Wallace C. Short-term intensive insulin therapy in newly diagnosed type 2 diabetes. Diabetes Care2004 May;27(5):1028-32.
51. Weng J, Li Y, Xu W, et al. Effect of intensive insulin therapy on beta-cell function and glycaemic control in patients with newly diagnosed type 2 diabetes: a multicentre randomised parallel-group trial. Lancet2008 May 24;371(9626):1753-60.
52. KDIGO clinical practice guideline for the care of kidney transplant recipients. Am J Transplant2009 Nov;9 Suppl 3:S1-155.
53. Joss N, Staatz CE, Thomson AH, Jardine AG. Predictors of new onset diabetes after renal transplantation. Clin Transplant2007 Jan-Feb;21(1):136-43.
54. Yale JF, Roy RD, Grose M, Seemayer TA, Murphy GF, Marliss EB. Effects of cyclosporine on glucose tolerance in the rat. Diabetes1985 Dec;34(12):1309-13.
55. Caro JF, Amatruda JM. Glucocorticoid-induced insulin resistance: the importance of postbinding events in the regulation of insulin binding, action, and degradation in freshly isolated and primary cultures of rat hepatocytes. J Clin Invest1982 Apr;69(4):866-75.
56. Luan FL, Steffick DE, Ojo AO. Steroid-free maintenance immunosuppression in kidney transplantation: is it time to consider it as a standard therapy? Kidney Int2009 Oct;76(8):825-30.
57. Davidson JA, Wilkinson A. New-Onset Diabetes After Transplantation 2003 International Consensus Guidelines: an endocrinologist's view. Diabetes Care2004 Mar;27(3):805-12.
58. Zambanini A, Newson RB, Maisey M, Feher MD. Injection related anxiety in insulin-treated diabetes. Diabetes Res Clin Pract1999 Dec;46(3):239-46.
59. Polonsky WH, Fisher L, Guzman S, Villa-Caballero L, Edelman SV. Psychological insulin resistance in patients with type 2 diabetes: the scope of the problem. Diabetes Care2005 Oct;28(10):2543-5.
60. Riddle MC, Rosenstock J, Gerich J. The treat-to-target trial: randomized addition of glargine or human NPH insulin to oral therapy of type 2 diabetic patients. Diabetes Care2003 Nov;26(11):3080-6.
61. Henry RR, Gumbiner B, Ditzler T, Wallace P, Lyon R, Glauber HS. Intensive conventional insulin therapy for type II diabetes. Metabolic effects during a 6-mo outpatient trial. Diabetes Care1993 Jan;16(1):21-31.
62. Johnston O, Rose CL, Webster AC, Gill JS. Sirolimus is associated with new-onset diabetes in kidney transplant recipients. J Am Soc Nephrol2008 Jul;19(7):1411-8.
63. International Expert Committee report on the role of the A1C assay in the diagnosis of diabetes. Diabetes Care2009 Jul;32(7):1327-34.
64. Report of the expert committee on the diagnosis and classification of diabetes mellitus. Diabetes Care2003 Jan;26 Suppl 1:S5-20.

65. Tura A, Kautzky-Willer A, Pacini G. Insulinogenic indices from insulin and C-peptide: comparison of beta-cell function from OGTT and IVGTT. Diabetes Res Clin Pract2006 Jun;72(3):298-301.
66. Katz A, Nambi SS, Mather K, et al. Quantitative insulin sensitivity check index: a simple, accurate method for assessing insulin sensitivity in humans. J Clin Endocrinol Metab2000 Jul;85(7):2402-10.
67. Pacini G, Mari A. Methods for clinical assessment of insulin sensitivity and beta-cell function. Best Pract Res Clin Endocrinol Metab2003 Sep;17(3):305-22.
68. Mari A, Pacini G, Murphy E, Ludvik B, Nolan JJ. A model-based method for assessing insulin sensitivity from the oral glucose tolerance test. Diabetes Care2001 Mar;24(3):539-48.

12. APPENDICES

Table 1. NPH Insulin Titration Regimen for Patients in Group A

Pre-dinner Capillary blood glucose	NPH dose initiation (IU/day)	NPH dose adjustment (IU/day)
≥ 240 mg/dl	14	Increase by 4
≥ 180 mg/dl	12	Increase by 4
≥ 140 mg/dl	10	Increase by 4
≥ 120 mg/dl	0	Increase by 2
100 to 119 mg/dl	0	Maintain the dose
80 - <100 mg/dl	0	Decrease by 4
60 - <80 mg/dl	0	Decrease by 8
< 60 mg/dl	0	Give ½ of previous dose

Table 2. Sliding Scale for Short Acting Insulin Regimen

Capillary blood glucose before meals and at bedtime (mg/dl)*	Short-acting insulin dose (IU)**
351 and above	16
301 to 350	12
251 to 300	8
201 to 250	4

*only high pre-lunch and at bedtime glucose levels will require short acting insulin coverage.

**given the next day at the meal at breakfast or dinner for the high lunchtime or bedtime glucose levels, respectively. Not to be administered at lunch or bedtime.

Table 3. Tacrolimus and Corticosteroids Dosing Schedule

	**Tacrolimus trough levels (ng/ml)**	**Corticosteroids (mg)***	**Corticosteroids (mg)****
At transplant (OR)		Methylprednisolone 500 mg intravenously	Prednisone 250 mg
Day 1		Prednisone 100 mg daily	Prednisone 125 mg daily
Day 2		Prednisone 80 mg daily	Prednisone 20 mg daily
Day 3		Prednisone 60 mg daily***	Prednisone 20 mg daily
Day 4		Prednisone 40 mg daily***	Prednisone 20 mg daily
Day 5 to 14	8 to 12	Prednisone 20 mg daily***	Prednisone 20 mg daily
Day 15 to 28		Prednisone 15 mg daily***	Prednisone 15 mg daily
Day 29 to 42		Prednisone 10 mg daily***	Prednisone 10 mg daily
Day 43 to 49		Prednisone 10 mg daily***	Prednisone 5 mg daily
Day 50 to 90		Prednisone 7.5 mg daily***	Prednisone 5 mg daily
Day 91 to 120		Prednisone 5 mg daily***	Prednisone 5 mg daily
Day 121 to 180	5 to 8	Prednisone 5 mg daily***	Withdrawal possible, but has to be reinforced by center practice in either all patients or none, regardless of glucose metabolism
Day 181 and beyond		Prednisone 2.5-5.0 mg daily***	

*EU protocol. **Barcelona protocol. ***Given orally.

In Austria: IV Methylprenisolone = Urbason®, IV Prednisone = SoluDacortin®, Oral Prednisone = Aprednislon®

Table 5. Sample Size and Power Calculations for Incidence of NODAT

Power 1 – β	**0.80**			**0.85**			**0.90**		
Difference *d*	0.10	0.15	0.20	0.10	0.15	0.20	0.10	0.15	0.20
Sample Size N	588	242	124	672	276	142	784	322	164

Table 6. Sample Size and Power Calculation for Hemoglobin A1c

Power 1 – β	**0.80**			**0.85**			**0.90**		
Difference *d*	0.3	0.5	0.7	0.3	0.5	0.7	0.3	0.5	0.7
Sample Size N	128	48	26	146	54	30	172	64	34

Table 4. Follow-up schedule for study visits and study related-procedure

	Screening & randomization	Weekly/biweekly (month 1 to 3)+	Month 3***	Month 6***	Month 12***	Month 24***
Eligibility Assessment	X					
Informed Consent	X					
Medical Record Consent	X		X	X	X	X
Contact Information	X	X	X	X	X	X
Demographic Information	X					
Eligibility Confirmation	X	X				
Comprehensive Clinical Assessment*	X		X	X	X	X
Fasting glycemia	X	X	X	X	X	X
Hemoglobin A1c	X		X	X	X	X
Fasting insulin/pro-insulin	X		X	X	X	X
Oral glucose tolerance test (OGTT)**				X	X	X
Microalbuminuria				X	X	X
Blood and/or urine samples for future studies				X	X	X
Recent medical events	X	X	X	X	X	X
Quality of life questionnaire	X			X	X	X

*Comprehensive Clinical Assessment includes (a) medical history; (b) clinical examination; and (c) routine laboratory studies. **For all patients, even if they are on any anti-diabetic medications (insulin or oral hypoglycemic agents), with medication not withdrawn before the test. The results of 6 month's OGTT will be blinded to study participants and investigators. ***Study windows: ±2 weeks. +Study windows: ±2 days.

Figure 1. Study Flow Chart

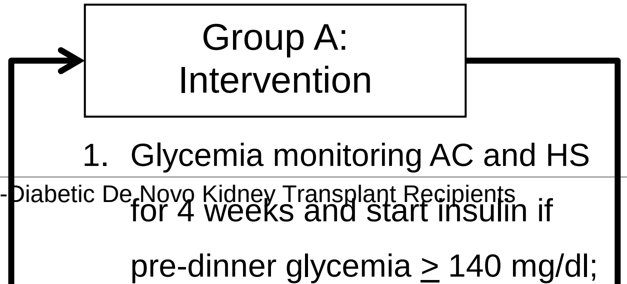

Supplement: S1 Protocol — Study protocol. Accessible online. (PDF) [file pone.0193569.s005.pdf]
