# Supplementary material for: A randomized controlled trial-based algorithm for insulin-pump therapy in hyperglycemic patients early after kidney transplantation
Source: PLoS One. 2018 Mar 8;13(3):e0193569. doi: 10.1371/journal.pone.0193569 (PMC5843249; doi:10.1371/journal.pone.0193569)
Supplement: S2 Protocol — Study protocol. Accessible online. (PDF) [file pone.0193569.s006.pdf]

Studie: SAPT-NODAT
Klinik: KIM III
EudraCT:

Treat-To-Target Trial of Continuous Subcutaneous, Sensor-Augmented Insulin-Pump Therapy in New-onset Diabetes after Transplantation (SAPT-NODAT): Efficacy and Safety of an Intensive Insulin Protocol in Renal Transplant Recipients Receiving a Tacrolimus-based Immunosuppression

Test drug (IMP)	*Insulin lispro® (Eli Lilly) applied in subcutaneous pumps*
Protocol authors	*Prof. Dr. Marcus Säemann, Dr. Manfred Hecking*
Academic investigators	*Manfred Hecking[1], Johannes Werzowa[1], Michael Haidinger[1], Markus Riegersperger[1], Walter Hörl[1], Ferdinand Mühlbacher[2], Susanne Rasoul-Rockenschaub[2], Johannes Pleiner[3], Marcus Säemann[1]*<br><br>*[1] Department of Internal Medicine III, Clinical Division of Dialysis and Transplantation, Medical University of Vienna, Austria*<br><br>*[2]Department of Surgery, Clinical Division of Transplantation, Medical University of Vienna, Austria*<br><br>*[3]Coordinating Center for Clinical Studies, Medical University of Vienna, Austria*
Document type	*Clinical study protocol*
Study phase	*II*
Document status	*Version 3.0*
Date	*July 22nd 2015*
Number of pages	*8*

Confidentiality Statement

The information contained in this document, especially unpublished data, is the property of the sponsor of this study, Department of Internal Medicine III, Division of Nephrology and Transplantation, Medical University Vienna. It is therefore provided to you in confidence as an investigator, potential investigator, or consultant, for review by you, your staff, and an Independent Ethics Committee or Institutional Review Board. It is understood that this information will not be disclosed to others without written authorization from the Department of Internal Medicine III, Division of Nephrology and Transplantation, Medical University Vienna, except to the extent necessary to obtain informed consent from those persons to whom the study drug may be administered.

SPONSOR, INVESTIGATOR, MONITOR AND SIGNATURES

Clinical investigator (AMG §§ 2a, 35,36)	*Doz. Dr. Marcus Säemann, Department of Internal Medicine III, Division of Nephrology and Dialysis, Medical University of Vienna, Austria*
Sponsor (AMG §§ 2a, 35,36)	*Medical University of Vienna, Spitalgasse 23, 1090 Wien, Austria*
Monitor (AMG §§ 2a, 35,36)	*Prof. Dr. Wilfred Druml, Department of Internal Medicine III, Division of Nephrology and Dialysis, Medical University of Vienna, Austria*
Clinical Trial Centers	*Department of Internal Medicine III Division of Nephrology and Dialysis and Department of Surgery, Division of Transplantation, Medical University of Vienna, Austria*

Doz. Dr. Marcus Säemann	________________ Signature	________________ Date
Prof. Dr. Wilfred Druml	________________ Signature	________________ Date
Prof. DDr. Walter Hörl	________________ Signature	________________ Date

PROTOCOL SYNOPSIS: SAPT-NODAT

Note:
In every aspect of patient inclusion, follow-up and care, other than insulin-administration, the SAPT-NODAT study will be conducted exactly in the same way as the ITP-NODAT study. However, while the ITP-NODAT study will randomize patients into *two* study arms, the SAPT-NODAT study will include patients in one study arm only, based on their call for transplantation at our center.

Specifically, for a fixed period, as determined by the Departments of Transplant Surgery and Nephrology, *all* non-diabetic patients at our center who are scheduled for transplantation (both deceased and living donors) will be informed and invited to participate in either ITP-NODAT or SAPT-NODAT. If, and only if, patients feel comfortable with *both* studies and would be willing to give informed consent for either, they will be randomized (1 to 2) for participation in either SAPT-NODAT or ITP-NODAT. If a patient is randomized for participation in ITP-NODAT, he/she will afterwards be randomized (1:1) into treatment or control group. If a patient is randomized in SAPT-NODAT, he will immediately receive the insulin pump (and continuous glucose sensor for safety, as stated below) by one of the academic investigators or the care-taking physicians on our intensive ward (13i3), where the patient is receiving his pre-transplantation dialysis. (For living-related kidney donation, a date will be fixed.)

TITLE	*Treat-To-Target Trial of Continuous Subcutaneous, Sensor-Augmented Insulin-Pump Therapy in New-onset Diabetes after Transplantation (SAPT-NODAT): Efficacy and Safety of an Intensive Insulin Protocol in Renal Transplant Recipients Receiving a Tacrolimus-based Immunosuppression*
ACRONYM	*SAPT-NODAT*
OBJECTIVES	**Primary Objective**<br>• *To demonstrate superiority of continuous subcutaneous sensor-augmented insulin-pump therapy (SAPT) with an insulin pump from Medtronic (Paradigm® Velo) for a period of approximately 3 months post-transplantation, and aiming for a pre-supper target capillary blood glucose level of 110 mg/dL against post-transplant hyperglycemia, <u>in comparison to conventional treatment</u>, and as evaluated by HbA1c at 3 months post-transplantation (comparison will be made against the simultaneously monitored control group of the*

	ITP-NODAT study [=arm B])<br>**Secondary Objectives**<br>• *To determine superiority of SAPT against post-transplant hyperglycemia, <u>in comparison to basal insulin treatment</u>, and as evaluated by HbA1c at 3 months post-transplantation (comparison will be made against the simultaneously monitored basal insulin treatment group of the ITP-NODAT study [=ITP-NODAT study arm A])*<br>• *To determine if SAPT with an insulin pump for a period of approximately 3 months post-transplantation, and aiming for a pre-supper target capillary blood glucose level of 110 mg/dL, can prevent intra-individually a clinically meaningful rise in HbA1c (≥0.5%), measured at 3 months post-transplantation*<br>• *To determine if HbA1c at 6, 12 and 24 months post-transplantation remains at the 3-months level, respectively the baseline level, intra-individually, even after intensive SAPT has been discontinued*<br><br>*(All of the following secondary endpoints will be evaluated against both, the simultaneously monitored control group of the ITP-NODAT study [=arm B] and the basal insulin treatment group of the ITP-NODAT study [=arm A]):*<br>• *Incidence of new-onset diabetes after transplantation, as diagnosed by an abnormal oral glucose tolerance on months 6, 12 and 24 months post- transplant*<br>• *HbA1c at 6, 12 and 24 months post-transplantation*<br>• *Change in HbA1c from baseline at 6, 12 and 24 months post-transplantation*<br>• *Glycemia profile under intensive insulin therapy (SAPT)*<br>• *Incidence of impaired fasting glycemia and impaired glucose tolerance 6, 12 and 24 months after transplantation.*<br>• *Pancreatic β-cell function at 6, 12 and 24 months after kidney transplantation, measured as insulin secretion during an OGTT in relation to the glucose stimulation (insulinogenic index – total and early phase)[1].*<br>• *Fasting insulin resistance (mostly liver) at 6, 12 and 24 months after kidney transplantation, measured by HOMA-R and by QUICKI (insulin sensitivity) from fasting (basal) glucose and insulin concentration[2,3].*<br>• *Dynamic insulin sensitivity (mostly muscle and adipose tissues) at 6, 12 and 24 months after kidney transplantation, measured by OGIS and ISIcomp from OGTT data[4].*<br>• *Renal function at 6, 12 and 24 months after kidney transplantation, measured by serum creatinine.*<br>• *Patient and graft survival 6, 12 and 24 months after kidney transplantation.*

DESIGN / PHASE	*Prospective, single-center, randomized, parallel group, controlled, phase II study.*
STUDY PLANNED DURATION	**First patient First visit** *1Q* 2013; **Last patient First visit** *1Q* 2016; **Last patient Last visit** *1Q* 2018
CENTER(S) / COUNTRY(IES)	*Department of Internal Medicine III, Division of Nephrology and Dialysis, Medical University of Vienna, Austria, Department of Surgery, Division of Transplantation, Medical University of Vienna, Austria*
PATIENTS / GROUPS	*34 patients in the SAPT-NODAT study, but 2 times 34 patients in the ITP-NODAT study (basal insulin treatment arm and standard of care control arm); hence altogether 3 groups*
INCLUSION CRITERIA	• *Adult patients with end stage renal disease undergoing kidney transplantation with a deceased or living donor kidney.*<br>• *Absence of diabetes prior to kidney transplantation, defined according to American Diabetes Association guideline (not on oral hypoglycemic agents or insulin with fasting glucose <126 mg/dL).*<br>• *Receiving standard triple immunosuppressive medications that include tacrolimus, mycophenolate mofetil or mycophenolic sodium and steroids.*<br>• *Capable of understanding the study and willing to give informed written consent for study participation.*
EXCLUSION CRITERIA	• *Patients with a diagnosis of diabetes mellitus prior to kidney transplantation, or receiving anti-diabetic medications, or having pre-transplant fasting glucose level equal or greater than 126 mg/dL on two occasions at least three days apart.*<br>• *Patients receiving an organ transplant other than kidney.*<br>• *Patients receiving an unlicensed drug or therapy within one month prior to study entry.*<br>• *Patients with history of hypersensitivity to injectable insulin.*<br>• *Patients with documented HIV infection.*
STUDY PERIODS	*From the approval of this application until completion of treatment for 34 patients the expected period of this study is 24 months.*
INVESTIGATIONAL DRUG	*Insulin lispro® from Eli Lilly), applied continuously with an insulin pump (Medtronic®)*
CONCOMITANT MEDICATION	**Allowed**
EFFICACY ENDPOINTS	**Primary**<br>• *HbA1c levels, in relative %, at 3 months. Superiority will be assumed if a statistically significant difference between the SAPT-treatment group versus the control group (from the ITP-NODAT study) can be determined.*<br>***Secondary***<br>• *HbA1c, in relative %, at 3, 6, 12 and 24 months post-*

	transplantation; The baseline measurement will also be subtracted from the 3-, 6-, 12-, and 24-months measurement (i.e. "3-months, 6-months, 12-months, and 24-months HbA1c minus baseline HbA1c"). For the determination of the intra-individual rise in HbA1c, the previously observed rise of 0.5±0.7 % (mean ± standard deviation) from baseline to 3 months in the TIP-study basal insulin treatment group will be judged to be clinically not meaningful, hence if the intra-individual rise in the SAPT-treatment group remains below that value, the rise in HbA1c will be considered to be not meaningful, clinically.<br>• *2h glucose ≥200 mg/dL, as by OGTT at 6, 12 and 24 months after transplantation (in comparison to the simultaneously monitored control group of the ITP-NODAT study [=arm B; control])*<br>• *Daily glycemia profile, through evaluation of all available glucose measurements*<br>• *Fasting glucose and 2h glucose at 6, 12 and 24 months after transplantation.*<br>• *Insulinogenic index during an OGTT at 6, 12 and 24 months after kidney transplantation1.*<br>• *HOMA-R and QUICKI at 6, 12 and 24 months after kidney transplantation[2,3].*<br>• *OGIS and ISIcomp at 6, 12 and 24 months after kidney transplantation[4].*<br>• *Serum creatinine at 6, 12 and 24 months after kidney transplantation*<br>• *Patient and graft survival at 6, 12 and 24 months after kidney transplantation*<br>• *Quality of life measures (mental component summary [MCS] and physical component summary [PCS] derived from the Kidney Disease Quality of Life Short Form (KDQoL-SF™) at 6, 12 and 24 months after kidney transplantation*
TOLERABILITY / SAFETY ENDPOINTS	*- Number of incidences of symptomatic hypoglycemia, confirmed by capillary blood glucose levels <60 mg/dL*
STATISTICAL METHODOLOGY	• **Primary Endpoint**<br>*HbA1c (%)*<br>• **Null and alternative hypotheses:**<br>***H_0*** *Treatment of hyperglycemia >140 mg/dl before supper with SAPT in renal transplant recipients is equal to conventional treatment in reducing the HbA1c (measured on day 90 after transplantation)*<br>***H_1****: Treatment of hyperglycemia >140 mg/dl before supper with SAPT is superior to conventional treatment in reducing the HbA1c (measured on day 90 after transplantation)*<br>**Type-I and -II errors - power.**<br>*α=0.05*<br>*ß=0.2*

	• **Statistical methodology**<br>*Two-sided t-test of HbA1c on day 90 after transplantation, two-sided t-test of number of days with hyperglycemia >140 mg/dl before supper, comparison of capillary blood glucose levels by ANOVA*<br>• **Sample size calculation**<br>*Based on a two-sided testing and an expected change in HbA1c of 10% with a standard deviation of 17%, an α=0.05 and a ß=0.2, a minimum sample size of 25 patients per group was determined. Additional 9 patients will be recruited in the pump arm according to the augmentation in the patient number for the ITP-NODAT study at our center (9 more patients included in the control and the basal insulin arm).*<br>• **Main analysis set**<br>*Per-protocol (efficacy) and intention to treat (ITT) for safety*<br>• **Other endpoints**<br>*(descriptive statistics).*

Further Details on the Study Procedure
(same as on p.3 [Note])

In every aspect of patient inclusion, follow-up and care, other than insulin-administration, the SAPT-NODAT study will be conducted exactly in the same way as the ITP-NODAT study. However, while the ITP-NODAT study will randomize patients into *two* study arms, the SAPT-NODAT study will include patients in one study arm only, based on their call for transplantation at our center.

Specifically, for a fixed period, as determined by the Departments of Transplant Surgery and Nephrology, *all* non-diabetic patients at our center who are scheduled for transplantation (both deceased and living donors) will be informed and invited to participate in either ITP-NODAT or SAPT-NODAT. If, and only if, patients feel comfortable with *both* studies and would be willing to give informed consent for either, they will be randomized (1 to 2) for participation in either SAPT-NODAT or ITP-NODAT. If a patient is randomized for participation in ITP-NODAT, he/she will afterwards be randomized (1:1) into treatment or control group. If a patient is randomized in SAPT-NODAT, he will immediately receive the insulin pump by one of the academic investigators or the care-taking physicians on our intensive ward (13i3), where the patient is receiving his pre-transplantation dialysis. (For living-related kidney donation, a date will be fixed.)

Insulin Pumps and Glucose Targets

The Paradigm® Veo insulin pumps (Medtronic) deliver insulin continuously. Patients can control when and at what rate their insulin is delivered. Insulin pump therapy allows patients to set one or several rates throughout the day and the night and to give a bolus or an insulin dose “on demand” when they eat. The (short-acting) insulin algorithms will be determined in collaboration with Dr. Andreas Thomas (Chief Scientific Officer in Germany), based on the glucose values obtained in the TIP-study control group[5]. The short-acting insulin we use will be Insulin lispro® (Eli Lilly). The pre-supper glucose target will be 110 mg/dL.

In addition to insulin delivery, the Paradigm® Veo insulin pumps (Medtronic) can react to glucose levels provided by a subcutaneous continuous glucose monitoring (CGM) system (also manufactured by Medtronic). Specifically, the pump will shut off insulin delivery upon a pre-determined setpoint of glucose, with the CGM sensor transmitting glucose readings to the insulin pump. In the present study, CGM will be used routinely in all patients, to increase study safety by possibly preventing hypoglycemic events.

References

1. Tura A, Kautzky-Willer A, Pacini G. Insulinogenic indices from insulin and C-peptide: comparison of beta-cell function from OGTT and IVGTT. Diabetes Res Clin Pract2006 Jun;72(3):298-301.

2. Katz A, Nambi SS, Mather K, et al. Quantitative insulin sensitivity check index: a simple, accurate method for assessing insulin sensitivity in humans. J Clin Endocrinol Metab2000 Jul;85(7):2402-10.

3. Pacini G, Mari A. Methods for clinical assessment of insulin sensitivity and beta-cell function. Best Pract Res Clin Endocrinol Metab2003 Sep;17(3):305-22.

4. Mari A, Pacini G, Murphy E, Ludvik B, Nolan JJ. A model-based method for assessing insulin sensitivity from the oral glucose tolerance test. Diabetes Care2001 Mar;24(3):539-48.

5. Hecking M et al., Manuscript accepted (JASN), presented at the ADA, And Diego, USA 2011 (oral presentation)
